# The Incidence of Contrast-Induced Nephropathy and the Need of Dialysis in Patients Receiving Angiography: A Systematic Review and Meta-Analysis

**DOI:** 10.3389/fmed.2022.862534

**Published:** 2022-04-27

**Authors:** Mei-Yi Wu, Wei-Cheng Lo, Yun-Chun Wu, Tsu-Chen Lin, Chun-Hung Lin, Mai-Szu Wu, Yu-Kang Tu

**Affiliations:** ^1^College of Public Health, Institute of Epidemiology and Preventive Medicine, National Taiwan University, Taipei, Taiwan; ^2^Division of Nephrology, Department of Internal Medicine, Shuang Ho Hospital, Taipei Medical University, New Taipei City, Taiwan; ^3^Division of Nephrology, Department of Internal Medicine, School of Medicine, College of Medicine, Taipei Medical University, Taipei, Taiwan; ^4^TMU Research Center of Urology and Kidney, Taipei Medical University, Taipei, Taiwan; ^5^Master Program in Applied Epidemiology, College of Public Health, Taipei Medical University, Taipei, Taiwan; ^6^Division of Urology, Department of Surgery, Chang Gung Memorial Hospital, Taoyuan City, Taiwan; ^7^Department of Orthopedics, Far Eastern Memorial Hospital, New Taipei City, Taiwan; ^8^School of Medicine, College of Medicine, Taipei Medical University, Taipei, Taiwan; ^9^Department of Dentistry, National Taiwan University Hospital and College of Medicine, Taipei, Taiwan

**Keywords:** administration route, contrast induced nephropathy, contrast media, meta-analysis, dialysis

## Abstract

**Objectives:**

The risk of dialysis following contrast exposure is unclear. We aimed to examine the overall risk of contrast induced nephropathy and the need of dialysis based on a systematic review with random-effects meta-analysis.

**Methods:**

We searched the electronic database including PubMed, Medline, Embase, and Cochrane Library from inception to 31 October, 2020 with predetermined search term to identify relevant studies. Observational studies investigating the association between contrast induced nephropathy after angiography and the need of dialysis were included, and summary risks were estimated. Two independent reviewers extracted the data, followed with random effects model to calculate the overall pooled incidence of contrast induced nephropathy and the need of dialysis after angiography. Subgroup-analysis and meta-regression were performed to assess heterogeneity of incidence across studies.

**Results:**

Of 2,243 identified articles, 259 met our inclusion criteria were included in the meta-analysis after screening. Pooled effect estimates had the following summary incidence proportion for contrast induced nephropathy after angiography: 9.06% (95% CI: 8.53–9.58%; derived from 120 studies) and 0.52% (95% CI: 0.37–0.70%; derived from 110 studies) for the need of dialysis, respectively. The stratified summary incidence proportion of contrast induced nephropathy after contrast administration *via* intra-arterial route was 9.60% (95% CI: 9.0–10.2%; derived from 106 studies) and was 0.6% (95% CI: 0.40–0.80%; derived from 100 studies) for the need of dialysis, respectively. Our meta-regressions found that the amount of contrast medium exposure was associated with contrast-induced nephropathy.

**Conclusion:**

The potential risk of dialysis needs to be communicated to patients undergoing procedures requiring contrast, especially *via* intra-arterial exposure.

**Systematic Review Registration:**

[https://reurl.cc/8Wrlry], identifier [CRD42020170702].

## Introduction

Prevalence of the end stage kidney disease (ESKD) related dialysis are expected to increase tremendously in the next decade (1). It is well documented that contrast-induced acute kidney injury (CI-AKI) is associated with developing chronic kidney disease (CKD). Even a single episode of CI-AKI can increase the risk of CKD progression (2, 3). The reported incidence of CI-AKI varies greatly according to the study population and the criteria of CI-AKI. It is reported that CI-AKI can develop in up to 55% of patients receiving contrast exposure during coronary angiography and result in 12.6% needing dialysis among the high-risk groups after percutaneous coronary intervention (4). The development of CI-AKI is associated with an increase in the length of hospital stay, higher care costs, and a greater risk of mortality (5).

Exposure to contrast medium, especially a large dose, has received substantial attention recently due to its potential associations with renal toxicity. However, data on the relationship between contrast medium exposure and the risk of the need of dialysis are scarce. Observational studies investigated the short- and long-term relationship between contrast exposure and the need of dialysis, but their diverse study designs yield wide variations in their results. The potential heterogeneity may have originated from the amount of contrast medium exposure, which would vary according to imaging techniques (percutaneous coronary intervention or angiography), types of contrast medium, routes of contrast medium administration, and populations with different comorbidities.

With the increasing use of interventional procedures such as coronary angiography, the incidence of CI-AKI is also rising. However, whether CI-AKI increases the need of renal replacement therapy (RRT) remains unclear. To address this knowledge gap, we did a systematic review and meta-analysis to estimate the incidence proportion of CI-AKI and the need of RRT after contrast medium exposure in patients receiving angiography. We also outlined the global map of the incidence proportion of CI-AKI and the need of dialysis.

## Materials and Methods

### Data Searches and Sources

Following the Preferred Reporting Items for Systematic Reviews and Meta-analysis (PRISMA) reporting guideline and protocol registration, we identified relevant studies through systematic searches of Medline, Pubmed, Embase, Ovid, and Cochrane library on 31, October, 2020. Search terms included those related to types of contrast exposure, the incidence of CI-AKI and the need of dialysis (see the Supplementary Material for details). No filter was applied to the study design. Screening the bibliographies of identified studies was also conducted. No restriction on the language of publications were imposed. After removing duplicate studies, titles and abstracts of the remaining studies were screened for eligibility; full texts of articles that met the inclusion criteria during the initial assessment were then retrieved. Two investigators (M-YW, T-CL) independently performed the screening of titles and abstracts as well as the evaluation of the full text of studies. Disagreements among the investigators were resolved by discussion.

### Study Selection and Design

We included studies that investigated the incidence of dialysis related to contrast induced nephropathy. Any procedures of radio-contrast exposure in angiography were considered, including coronary angiography. Studies on all types of contrast-induced nephropathy were included, but animal studies were excluded. All review and data extraction processes were in accordance with our prespecified review protocol (PROSPERO registration CRD42020170702). The main outcome was the pooled incidence proportion of CI-AKI after angiography. For all studies, the proportion of patients with CI-AKI was calculated as the number of patients with CI-AKI divided by the total number of patients undergoing angiography. We estimated the pooled incidence proportion of the need of dialysis after angiography.

### Data Extraction

Data extraction was performed for all studies by two investigators. Extracted data included the publication details, study design, setting, population size and demographics, time frame, definition of contrast-induced nephropathy, routes of contrast medium administration, procedures of radio-contrast exposure, baseline renal function, and the confounders that were adjusted for. The outcome measure required for inclusion was assessment of incidence of contrast-induced nephropathy or the need of dialysis after contrast medium exposure. We extracted data on the estimates of incidence proportion [and 95% confidence intervals (CIs)] of incident CI-AKI and dialysis. Two investigators (M-YW, T-CL) independently extracted the information and estimations of each study, and a third investigator (C-HL) resolved disagreements independently.

### Quality Appraisal

In the absence of any validated quality assessment tools for incidence proportion investigation, two reviewers (M-YW, T-CL) independently evaluated the quality of each included studies using the modified Newcastle–Ottawa-Quality Assessment Scale (NOS) and the Joanna Briggs Institute Critical Appraisal tools (6, 7). The evaluation of methodological quality across studies was based on the following factors: representativeness of the population, clear case definition relevant to our inclusion criteria, and high response rate with sufficient follow-up time. We assigned each quality domain as “high quality” or “low quality.” In the event of insufficient details reported in a study, we scored the quality as “uncertain.” Disagreements were resolved first by discussion and then by consulting a third author (W-CL) for arbitration.

### Statistical Analysis

We undertook the random-effects meta-analysis to estimate the incidence proportion of CI-AKI and the need of RRT. The Stata command, “metaprop,” was used to pools incidence proportions with the score statistic and the exact binomial method (8). The method provided appropriate ways of combining proportions close to the margins by using the Freeman–Tukey Double Arcsine Transformation to compute 95% confidence intervals. Cochran Q test with *P* < 0.10 and I2 statistic were used to evaluate between-study heterogeneity. Subgroup-analysis was performed to assess estimates by study designs, intervention procedures and administration route. Meta-regression was used to assess if heterogeneity of incidence proportions across studies depended on population characteristics, comorbidities, and follow-up days for RRT and year of publication. Data analysis was performed with Stata 16.1 (College Station, TX, United States).

### Publication Bias

Since conventional funnel plots could be inaccurate for meta-analyses of proportion studies with extreme proportional outcomes, we therefore assessed publication bias by using the Doi plot and Luis Furuya-Kanamori asymmetry index (LFK index) (9, 10). When a symmetrical Doi plot is presented, no publication bias is expected. A LFK index within ±1 was considered as non-asymmetry, between ±1 and ±2 as minor asymmetry, and above ±2 as major asymmetry. To better describe the statistical properties for meta-analysis, we also transformed the proportion to the log odds scale and log standard error. Consider a study in which *r* out of *n* patients were observed to have an event, leading to a proportion of *r/n*. The associated log odds is ln*[r/(n–r)]* with standard error sqrt*[1/r* + *1/(n–r)]*, which were used to evaluate the potential publication bias (9). Additionally, Egger’s test was performed to detect the asymmetry (*p*-value < 0.1 indicates the present of asymmetry) (11).

## Results

### Pooled Effect Estimates

**FIGURE 1:**
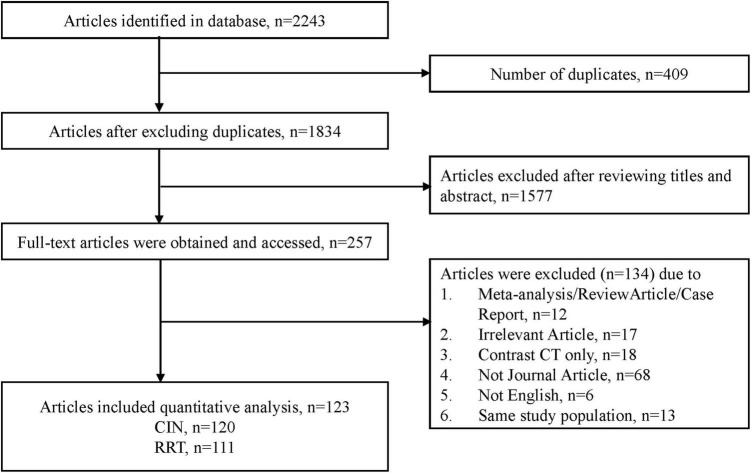
Flow diagram of articles considered for inclusion.

The details of our literature search are shown in Figure 1. We identified 329 potentially eligible articles after excluding duplications and title and abstract screening. Eventually, 230 articles were included in quantitative analysis after excluding review article, irrelevant articles and receiving contrast CT only. Of the 230 articles, 120 studies with 974,898 participants reported the numbers of patients with contrast-induced nephropathy and 111 studies with 858,305 participants reported the numbers of patients requiring dialysis (Figure 1). The pooled incidence proportion of CIN was 9.06% (95% CI: 8.53–9.58%; I2: 99.3%). The pooled incidence proportion of the need of dialysis was 0.52% (95% CI: 0.37–0.70%; I2: 96.1%). The subgroup analysis revealed that the incidence proportion of CIN in randomized-controlled trials (RCTs), prospective and retrospective studies were 14.97% (95% CI: 12.82–17.12%; I2: 86.14%; ranged from 0 to 50.0% with median of 13.32%), 8.65% (95% CI: 7.63–9.67%; I2: 98.48%; ranged from 0 to 35.59% with median of 9.56%), and 7.88% (95% CI: 7.15–8.61%; I2: 99.61%; ranged from 0 to 65.0% with median of 8.75%), respectively (Table 1 and Supplementary Figure 1). The subgroup analysis showed that the incidence proportion of the need of dialysis in RCTs, prospective and retrospective studies were 1.00% (95% CI: 0.40–1.79%; I2: 76.4%; ranged from 0 to 35.0% with median of 0.63%), 0.83% (95% CI: 0.37–1.42%; I2: 90.88%; ranged from 0 to 16.72% with median of 0.51%), and 0.39% (95% CI: 0.22–0.59%; I2: 97.84%; ranged from 0 to 17.11% with median of 0.39%), respectively (Table 1 and Supplementary Figure 2). The subgroup analysis revealed the incidence proportion of CIN in studies with heart-related, not-heart-related, and other procedures were 9.90% (95% CI: 9.29–10.50%; I2: 99.44%), 5.54% (95% CI: 4.00–7.07%; I2: 95.29%), and 8.01% (95% CI: 5.40–10.62%; I2: 90.71%), respectively (Table 1 and Supplementary Figure 3). The subgroup analysis revealed the incidence proportion of the need of dialysis in studies with heart-related, non-heart related, and other procedures were 0.59% (95% CI: 0.40–0.80%; I2: 96.70%), 0.29% (95% CI: 0.04–0.70%; I2: 70.99%), and 0.44% (95% CI: 0.12–0.89; I2: 44.17%), respectively (Table 1 and Supplementary Figure 4). The subgroup analysis showed that the incidence proportion of CIN in studies with intra-arterial, intra-venous, and intra-arterial/intra-venous were 9.6% (95% CI: 9.0–10.2%; I2: 99.39%), 2.6% (95% CI: 1.1–4.1%; I2: 81.96%), and 11.0% (95% CI: 8.3–13.7%; I2: 78.94%), respectively (Table 1 and Supplementary Figure 5). For the need of dialysis, the incidence proportion in studies with intra-arterial, intravenous, and intra-arterial/intravenous were 0.6% (95% CI: 0.4–0.8%; I2: 96.34%), 0.00% (95% CI: 0.0–0.30%; I2: 16.79%), and 0.8% (95% CI: 0.3–1.7%; I2: 79.3%), respectively (Table 1 and Supplementary Figure 6). The summary incidence proportion for contrast induced nephropathy before 2010 was 9.8% (95% CI: 8.9–10.6%; derived from 65 studies) and 8.7% (95% CI: 7.9–9.4%; derived from 55 studies) after 2010, respectively (Supplementary Figure 7). The summary incidence proportion for the need of dialysis before 2010 was 0.8% (95% CI: 0.5–1.1%; derived from 60 studies) and 0.4% (95% CI: 0.2–0.6%; derived from 50 studies) after 2010, respectively (Supplementary Figure 8).

**TABLE 1:** Summary of overall and stratified meta-analysis results.

Outcome	Strata	Number of studies	Incidence proportion	(95% CI)	I^2^
CIN	**Study design**				
	RCT (placebo or control group)	42	14.97%	(12.82%, 17.12%)	86.14%
	Prospective study	36	8.65%	(7.63%, 9.67%)	98.48%
	Retrospective study	42	7.88%	(7.15%, 8.61%)	99.61%
	Angiography procedure				
	Heart-related	94	9.90%	(9.29%, 10.50%)	99.44%
	Non-heart-related	14	5.54%	(4.00%, 7.07%)	95.29%
	Others	12	8.01%	(5.40%, 10.62%)	90.71%
	**Administration route**				
	Intra-arterial	106	9.61%	(9.04%, 10.18%)	99.39%
	Intravenous	8	2.63%	(1.12%, 4.14%)	81.96%
	Intra-arterial and Intravenous	6	11.00%	(8.27%, 13.73%)	78.94%
	Total	120	9.06%	(8.53%, 9.58%)	99.33%
RRT	**Study design**				
	RCT (placebo or control group)	40	1.00%	(0.40%, 1.79%)	76.40%
	Prospective study	30	0.83%	(0.37%, 1.42%)	90.89%
	Retrospective study	41	0.39%	(0.22%, 0.59%)	97.84%
	**Angiography procedure**				
	Heart-related	88	0.59%	(0.40%, 0.80%)	96.70%
	Non-heart-related	12	0.29%	(0.04%, 0.70%)	70.99%
	Others	11	0.44%	(0.12%, 0.89%)	44.17%
	**Administration route**				
	Intra-arterial	100	0.56%	(0.39%, 0.76%)	96.34%
	Intravenous	7	0.02%	(0.00%, 0.27%)	16.79%
	Intra-arterial and Intravenous	4	0.83%	(0.25%, 1.71%)	79.30%
	Total	111	0.52%	(0.37%, 0.70%)	96.07%

*CIN, contrast-induced nephropathy; RRT, renal replacement therapy; RCT, randomized controlled trial.*

As depicted in Table 2, univariable meta-regression analysis revealed that study design, angiography procedures, administration route, and representativeness of study population were significantly associated with heterogeneity of CIN incidence estimates, and median age and amount of contrast medium exposure approached the borderline of significance. In the multivariable meta-regression model, amount of contrast medium exposure, and representativeness of study population remained significantly associated with heterogeneity of results. An increase of one unit in contrast medium exposure leads to a rise in CIN incidence by 0.043%, after adjusting other important covariates. Table 3 presents the meta-regression analyses of potential source of heterogeneity for incidence proportion of RRT. We included the covariates that significantly associated with heterogeneity of incidence proportion of RRT in univariable model into multivariable meta-regression model. We found that representativeness of study population remained associated with heterogeneity (*p*-value: 0.001).

**TABLE 2:** Univariable and multivariable meta-regression analyses of potential source of heterogeneity for incidence proportion of contrast-induced nephropathy.

		Univariate model				Multivariate model^$^		
			
Moderators	# of studies	Coef.	95% CI	*P*-value	Adjusted *R*^2^	Coef.	95% CI	*P*-value
Study design^1^	120	–0.060	(–0.101, –0.020)	0.004	7.10%	–0.005	(–0.058, 0.047)	0.837
Angiography procedure^2^	120	–0.065	(–0.111, –0.020)	0.005	6.78%	–0.062	(–0.134, –0.009)	0.086
Administration route^3^	120	–0.065	(–0.124, –0.006)	0.03	4.02%	–0.007	(–0.115, –0.101)	0.896
Publication year	120	–0.002	(–0.004, 0.001)	0.2	–0.03%			
Median age	110	–0.003	(–0.006, 0.000)	0.07	4.29%	–0.003	(–0.007, 0.002)	0.229
Proportion of male	110	0.156	(–0.036, 0.348)	0.1	1.27%			
Incidence of treated ESRD	85	–0.000	(–0.000, 0.000)	0.2	0.66%			
Baseline creatinine	90	–0.001	(–0.004, 0.002)	0.6	–1.32%			
Proportion of patients with eGFR < 60 mL/min	47	–0.011	(–0.075, 0.053)	0.7	–2.08%			
Amount of contrast medium exposure (mL)	81	0.000	(–0.000, 0.001)	0.07	3.56%	0.000	(0.000, 0.001)	0.02
Days between CM administration and creatinine measurements	98	–0.001	(–0.007, 0.005)	0.7	–1.49%			
RRT follow-up time	52	–0.000	(–0.000, 0.000)	0.7	–2.92%			
Representativeness^4^	120	–0.029	(–0.049, —0.009)	0.004	6.05%	–0.043	(–0.073, –0.012)	0.007

*^1^Observational study vs. RCT;*

*^2^Computed tomography angiography and others vs. coronary angiography;*

*^3^Intravenous and others vs. intra-arterial;*

*^4^Score of representativeness (high quality: 2 points; uncertain: 1 point; low quality: 0 point; see Supplementary Material for more details).*

*^$^Adjusted R^2^ for multivariate model = 14.58%.*

**TABLE 3:** Univariable and multivariable meta-regression analyses of potential source of heterogeneity for incidence proportion of renal replacement therapy.

		Univariate model				Multivariate model^$^		
			
Moderators	# of studies	Coef.	95% CI	*P*-value	Adjusted *R*^2^	Coef.	95% CI	*P*-value
Study design^1^	111	–0.000	(–0.006, 0.005)	0.9	–3.15%			
Angiography procedure^2^	111	–0.001	(–0.007, 0.004)	0.6	–10.18%			
Administration route^3^	111	–0.002	(–0.009, 0.005)	0.56	–8.62%			
Publication year	111	–0.000	(–0.000, 0.000)	0.4	0.01%			
Median age	102	0.000	(–0.000, 0.001)	0.8	–4.67%			
Proportion of male	105	0.003	(–0.022, 0.028)	0.4	–3.57%			
Incidence of treated ESRD	82	0.000	(–0.000, 0.000)	0.5	–8.54%			
Baseline creatinine	83	–0.000	(–0.000, 0.000)	0.7	–3.75%			
Proportion of patients with eGFR < 60 mL/min	43	0.009	(0.002, 0.017)	0.02	14.9%	–0.002	(–0.012, 0.008)	0.7
Amount of contrast medium exposure (mL)	75	–0.000	(–0.000, 0.000)	0.9	–6.50%			
Days between CM administration and creatinine measurements	90	0.000	(–0.001, 0.001)	0.9	–7.56%			
RRT follow-up time	54	0.000	(–0.000, 0.000)	0.6	–5.09%			
Representativeness^4^	111	–0.004	(–0.006, -0.002)	0.001	53.02%	–0.005	(–0.010, -0.001)	0.02

*^1^Observational study vs. RCT;*

*^2^Computed tomography angiography and others vs. coronary angiography;*

*^3^Intravenous and others vs. intra-arterial;*

*^4^Score of representativeness (high quality: 2 points; uncertain: 1 point; low quality: 0 point; see Supplementary Material for more details).*

*^$^Adjusted R^2^ for multivariate model = 68.47%.*

**FIGURE 2:**
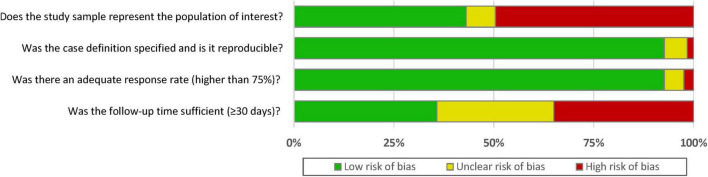
Risk of bias assessment.

A summary of the quality assessment (risk of bias assessment) of included studies is presented in Figure 2. Most studies [96 (78%)] had an overall rating of good or fair quality, while 27 (22%) studies were rated as poor (Supplementary Figure 1). For representativeness of the target population, 43.1% of included studies were rated as low risk of selection bias. On the other hand, there was 93% of included studies reported clear criteria to diagnosis CIN event and 93% of included studies had an adequate response rate during the follow-up. However, only 36% of included studies reported sufficient follow-up time (≥ 30 days).

### Global Distribution

**FIGURE 3:**
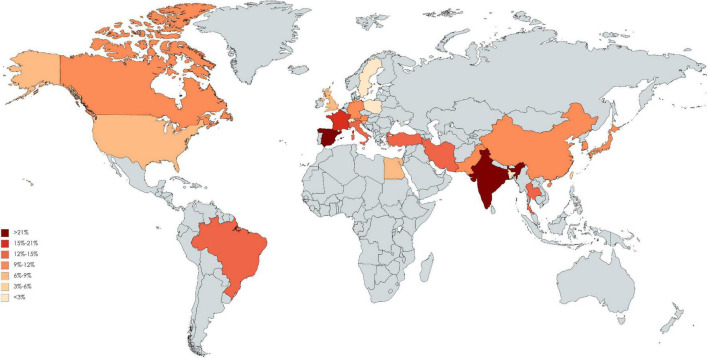
Global incidence proportion of acute kidney injury after angiography.

**FIGURE 4:**
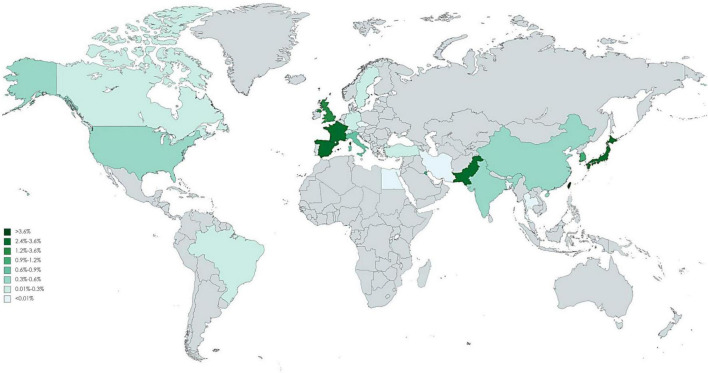
Global incidence proportion of angiography-related contrast induced nephropathy requiring dialysis.

Of 123 included studies, 36 studies were from Europe, 37 were from Asia, 2 were from South America (Brazil), 46 were from North America, and 2 were from Africa (Egypt). The pooled incidence proportion of CIN and RRT according to geographic location of the study is provided in Figures 3, 4. The countries with the highest CIN incidence proportion were Spain (31.1%; 95% CI, 5.4-56.8%), India (28.6%; 95% CI, 9.1-48.2%), and France (20.1%; 95% CI, 17.9-22.3%). Countries with the lowest CIN incidence proportion were Kuwait (1.02%; 95% CI, 0.03-5.55%), Switzerland (1.37%; 95% CI, 0.76-1.99%), and Sweden (1.51%; 95% CI, 1.41-1.61%). The highest incidence proportion of RRT occurred in Taiwan (12.1%; 95% CI, 0-48.7%). However, the heterogeneity was high even stratified by study country, and thus these results should be interpreted cautiously.

### Publication Bias

The visual asymmetry of the Doi plot suggested potential publication bias, where smaller studies reported higher incidence proportion estimates (Supplementary Figures 9, 10). When stratifying the results by study design, major asymmetry suggesting publication bias were presented for incidence proportion of CIN in prospective and retrospective studies, while no asymmetry in RCTs. The Egger’s test shows compatible signs with publication bias, *p*-value was 0.014, 0.051, and 0.092, respectively. Minor and major asymmetry were detected for incidence proportion of the need of dialysis in RCTs, prospective and retrospective studies with LFK indexes of -1.33, -1.34, and 2.46, respectively (Egger’s test was *p* = 0.076, 0.976, and 0.062, respectively). To observe the potential effect of small study, we therefore excluded the studies with sample size smaller than 100 participants and performed sensitivity analysis (Supplementary Figures 11, 12). In general, the result was consistent with main analysis. The incidence proportion of CIN in RCTs, prospective and retrospective studies were 12.35, 8.92, and 7.69%, respectively, while the incidence proportion of the need of dialysis in RCTs, prospective and retrospective studies were 0.59, 0.78, and 0.44%, respectively.

## Discussion

This systematic review and meta-analysis found that the incidence proportion of CIN after the contrast medium exposure was 9% and that of kidney failure requiring RRT was 0.5%. The stratified incidence proportion of CIN after coronary angiography-related exposure is 9.90 and 0.59% for the need of dialysis. Our study also confirmed previous findings that the risk of acute kidney injury was higher after intra-arterial than after intravenous contrast medium administration (12). However, we noted that the need of dialysis for patients, who are given intravenous contrast medium, was very low. We also observed that the incidence proportion of CIN seemed to be decreasing, and a similar trend was also observed for the need of dialysis. Several approaches to the prevention of CIN have been investigated (13), but the results were not promising except for the use of preprocedural hydration as the standard of care for prophylaxis.

Previous studies on CIN focused on short-term renal dysfunction, but our systematic review evaluated the severe events, such as the need of dialysis after CIN. We expect our findings to increase the awareness of the harmful effects of the contrast medium exposure on the risk of CIAKI and dialysis, which is an issue attracting attention from the nephrology community. Because of the high incidence of vascular diseases in patients with CKD, the number of percutaneous coronary intervention procedures being performed has been increasing over past years (14). Our meta-analyses found that for a substantial number of patients, their renal function after exposure to contrast medium gets worsened to the extent that they require dialysis. Identifying patients who are at higher risks for CIAKI and dialysis is therefore critical.

Contrast-induced nephropathy after the intra-arterial administration is well documented, but its occurrence after intravenous administration is controversial. A meta-analysis contained a total of 19,563 patients to evaluate the serum creatinine changes following contrast-enhanced CT imaging have reported that CIN occurred in 6% of patients after the contrast-enhanced CT and 0.06% of those receiving RRT. Our study focuses on angiography which received contrast medium from intravenous and intra-arterial injection. The incidence proportion of CIN after intra-arterial administration was shown to be higher than that *via* the intravenous administration. Intra-arterial administration requires arterial access, and atheroembolic complications and population-specific risk factors were believed to be associated with the higher incidence of CI-AKI. A recent randomized trial showed intravenous contrast medium administration had a lower rate of CI-AKI compared with that *via* the intra-arterial administration (15).

Contrast medium exposure predisposes susceptible individuals to experiencing deterioration of renal function, which in turn, elevates the risk of requiring dialysis. This remains a crucial concern. A large variation in CI-AKI incidence after percutaneous coronary intervention was reported. These inconsistencies may result from study design, the follow-up duration, the heterogeneity of contrast medium exposures, or the adjustments for different confounders. CI-AKI is supposed to be common, morbid, and costly. A large study of more than 1.3 million patients on the National Cardiovascular Data Registry CathPCI Registry highlights the importance to reducing the contrast medium use to reduce CI-AKI (16). However, few studies have reported association between contrast medium exposure and the need of dialysis.

Contrast-induced acute kidney injury was associated with an increased risk of need for dialysis, CKD, and mortality in those undergoing percutaneous coronary intervention for ST-elevation myocardial infarction (17, 18). A study proposed the importance of post-discharge follow-up because CI-AKI post PCI is associated with increased risk of death, myocardial infarction, and recurrent kidney injury post discharge (19). Patients with CKD were reported to be have a high rate of CI-AKI but the need of dialysis both in-hospital or on follow-up is infrequent (20). In agreement with the previous study, our meta-regression results showed the patients with CKD and lower baseline renal function have a higher incidence rate of CIN (18). Our meta-regression results further concluded the CKD and lower baseline renal function have a higher incidence rate of the need of dialysis.

The studies identified in this systematic review present widely differing incidence estimates in CIN. Although the socioeconomic factors, genetic susceptibility, and health system may relate to the underlying difference between countries, the heterogeneity remains high in stratification analysis. Thus, these results should be interpreted cautiously. The identified literature had a geographical focus on North America, European, and part of Asia countries. Beyond this region, the situation on the rest of the world remains largely unknown. Of note, these findings do not indicate that CIN is non-existent elsewhere. The scarcity of data may be related to the weak health care systems, the absence of surveillance systems, or limited or no report documented the relevant information. Although in a large cohort study, race was not associated with the development of CI-AKI and CKD post angiography or intervention, the Caucasian patients had significant lower rate of initiation of dialysis (21). In our global mapping showed there are several hot areas of elevated incidence of CI-AKI including India, Spain and France, which is consistent with the prior study. The renal impacts of contrast medium have been a serious challenge because it is unavoidable to receive it if coronary artery disease is highly suspected. A cross-section study indicated there is a large variation in use of contrast medium volumes among physicians (16). We should stress the influence of the need of dialysis and restrict the amount of contrast volumes in high-risk patients. Potential biological mechanisms include the potential translocation of ultrafine particles into circulation, which leads to coagulation or fibrinolytic dysfunction. Nonetheless, studies included in this meta-analysis generally indicate that contrast medium exposure play certain role to affect renal health, thus warranting our attention in high-risk groups. Data on the optimal strategy to reduce CI-AKI was conflicting, several potential therapies were ineffective in patients with CKD (22). The task of confronting the increasing global prevalence of ESKD is challenging. In addition to conventional risk factors of CKD, contrast medium may be an under-recognized factor causing the rising trend of CKD. Patients with CKD and their caregivers should be informed of the adverse effects of contrast medium on kidney health.

Several limitations of our study should be noted. First, our analysis included participants with a range of medical conditions, such as stroke, diabetes, CVD, and impaired renal function, participants with different profiles of underlining conditions may have different vulnerability of CI-AKI. Those variations increased the heterogeneity within our findings, but we still believe that it is worth of the efforts to estimate the impact of CI-AKI globally. Second, there are different observation periods of renal outcomes in the included studies on the relationship between contrast medium exposure and the need of dialysis. Moreover, differences exist in the estimation of renal function and the accurate diagnosis of CI-AKI. Third, retrospective studies searched medical records to identify potential cases of CI-AKI, and this may yield under-estimation of risks if some cases were not recorded in the health care system. Finally, considerable differences also exist between studies with regarding to participant characteristics, the amount of contrast medium, follow-up period, and outcome assessment. Although multivariable meta-regression model showed that the increase in the amount of contrast medium was significantly associated with the increase in the incidence proportion of CI-AKI; however, the residual heterogeneity remains high. Without individual patient data, we cannot rule out the ecological bias when study-level covariates were used. Further studies are required to identify subgroups of patients who are most susceptible to the harmful effects of contrast medium exposure.

In this systematic review and meta-analysis, we have provided updated evidence on the association between exposure to contrast medium and the increased risk of CI-AKI and the need of dialysis, especially *via* intra-arterial route. The potential risk of dialysis needs to be communicated to patients undergoing diagnostic and/or interventional procedures requiring contrast medium. To reduce the impact of ESKD on patients’ health and quality of life, developing strategies to minimize renal toxicity of contrast medium exposure is a priority.

## Data Availability Statement

The original contributions presented in the study are included in the article/Supplementary Material, further inquiries can be directed to the corresponding author.

## Author Contributions

M-YW and W-CL: had full access to all data in this study, took responsibility for the integrity of the data, accuracy of the related analysis, and statistical analysis. M-YW, M-SW, and Y-KT: concept and design. M-YW, W-CL, T-CL, and C-HL: literature search and data analysis and interpretation. M-YW, W-CL, and Y-KT: drafting of the manuscript. W-CL, Y-CW, and M-SW: administrative, technical, and material support. Y-KT: supervision. All authors critical revision of the manuscript on important intellectual content.

## Conflict of Interest

The authors declare that the research was conducted in the absence of any commercial or financial relationships that could be construed as a potential conflict of interest.

## Publisher’s Note

All claims expressed in this article are solely those of the authors and do not necessarily represent those of their affiliated organizations, or those of the publisher, the editors and the reviewers. Any product that may be evaluated in this article, or claim that may be made by its manufacturer, is not guaranteed or endorsed by the publisher.
