# Supplementary material for: The Incidence of Contrast-Induced Nephropathy and the Need of Dialysis in Patients Receiving Angiography: A Systematic Review and Meta-Analysis
Source: Front Med (Lausanne). 2022 Apr 27;9:862534. doi: 10.3389/fmed.2022.862534 (PMC9091353; doi:10.3389/fmed.2022.862534)
Supplement: Supplementary file 1 [file Data_Sheet_1.docx]

Supplementary Material

# Search strategy

- We performed a systematic review of the literature according to the PRISMA (Preferred Reporting Items for Systematic Review and Meta-Analysis) guidelines.
- We searched the EMBASE, PUBMED, MEDLINE, and the Cochrane Library for articles published before 31 Oct, 2020. The search term used were as following:
- Dialysis-
- Emtree- “dialysis”, “renal replacement therapy”
- MeSH term- “renal dialysis”, “Hemodialysis, Home”, “dialysis”, “Peritoneal Dialysis, Continuous Ambulatory”, “Peritoneal Dialysis”, “renal replacement therapy”
- Keywords- “dialysis”, “renal replacement therapy”, “hemodialysis”, “peritoneal dialysis”, “rrt”
- Contrast induced nephropathy
- Emtree- “contrast induced nephropathy”
- MeSH term- none
- Keywords-
- “contrast induced nephropathy”, “contrast induced acute kidney injury”, “contrast induced nephrotoxicity”, “contrast induced renal failure”, “contrast induced renal dysfunction”, “contrast agent induced nephropathy”, “contrast agent induced acute kidney injury”, “contrast agent induced nephrotoxicity”, “contrast agent induced renal failure”, “contrast agent induced renal dysfunction”, “contrast medium induced nephropathy”, “contrast medium induced acute kidney injury”, “contrast medium induced nephrotoxicity”, “contrast medium induced renal failure”, “contrast medium induced renal dysfunction”, “contrast media induced nephropathy”, “contrast media induced acute kidney injury”, “contrast media induced nephrotoxicity”, “contrast media induced renal failure”, “contrast media induced renal dysfunction”, “CIN”, “CIAKI”, “CI-AKI”

# Quality appraisal

The quality assessment of the selected studies was performed by using a modifiable appraisal form according to the Newcastle–Ottawa-Quality Assessment Scale and the Joanna Briggs Institute Critical Appraisal tools to cover the major concern of potential risk of biases. Two reviewers (MYW, TCL) independently evaluated the quality of each included studies for four following questions:

(1) Was the study’s target population a close representation of the general population in relation to relevant variables?

(2) Was the case defined in a standard, valid, and reliable way for all participants?

(3) Was the response rate adequate, and if not, was the low response rate managed appropriately?

(4) Was the follow‐up time sufficient to observe the outcome?

Studies may report how recruitment or sampling methods was performed. If study excluded the individuals with specific health condition or only included patients with specific disease outcomes (e.g., stroke, diabetes, or CKD), the study quality in the representativeness of the population was defined as ‘uncertain’ or ‘low quality’ (For RCT study design, we only look at the control or placebo group). The primary outcome of this systematic review and meta-analysis was contrast induced nephropathy (CIN). The most widely accepted definition of CIN is an increase of 25% or more, or an absolute increase of 0.5 mg/dL (44 μmol/L) or more in serum creatinine from baseline values, at 24–72 hours following exposure to contrast media. If included study has clearly described the definition of CIN as above criteria, it will be graded as ‘high quality’. The response rate that was higher than 75% was defined as adequate (high quality), otherwise inadequate (low quality). The follow‐up time more than 30 days was defined as sufficient (high quality), otherwise insufficient (low quality). According to above criteria, we assigned each quality domain as ‘high quality’ with 2 points or ‘low quality’ with 0 point. In the event of insufficient details reported in a study, we graded the quality as ‘uncertain’ with 1 point. Disagreements were resolved first by discussion and then by consulting a third author (WCL) for arbitration.

# Supplementary Figures and Tables

## Supplementary Figures


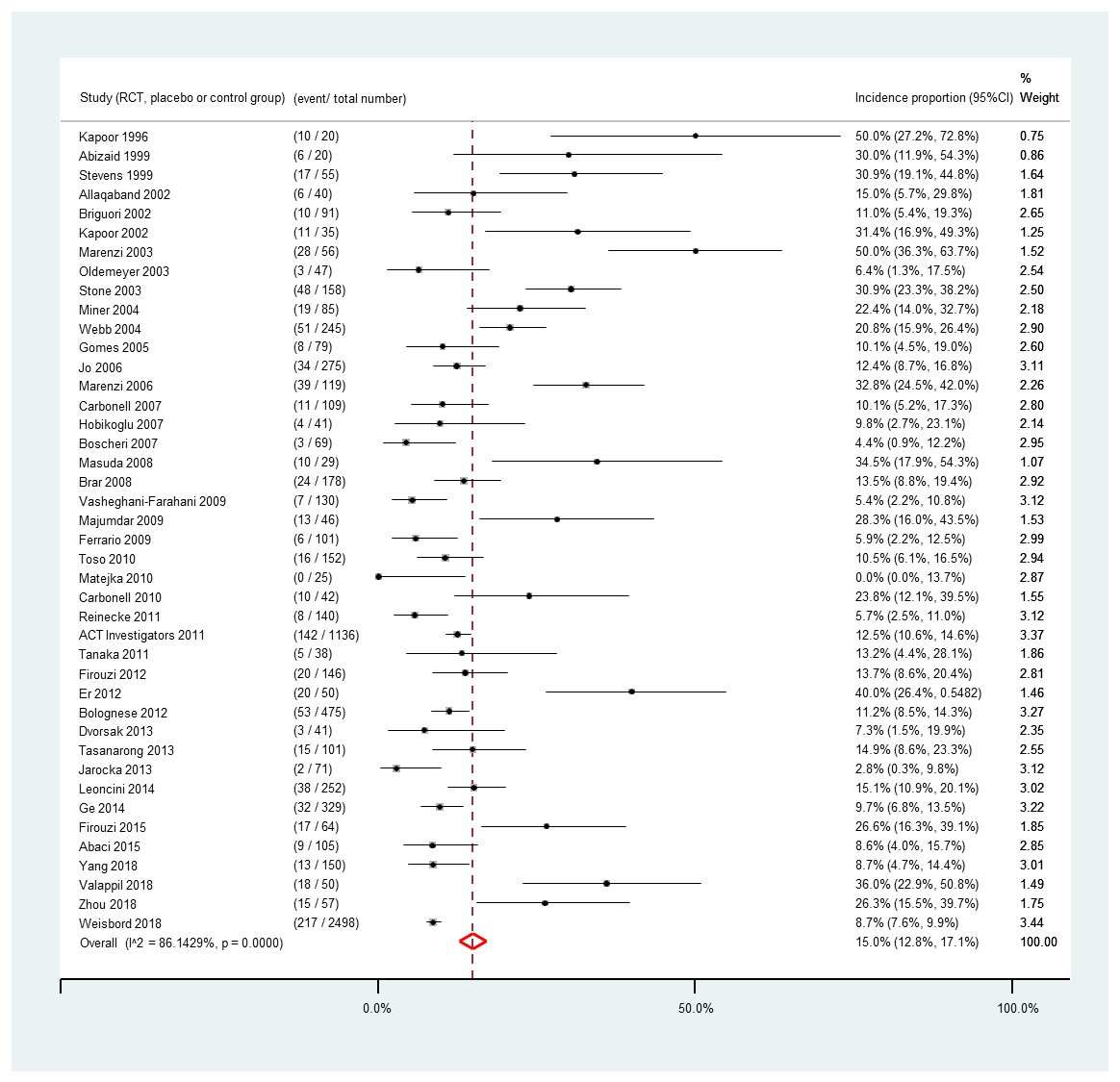
(A)


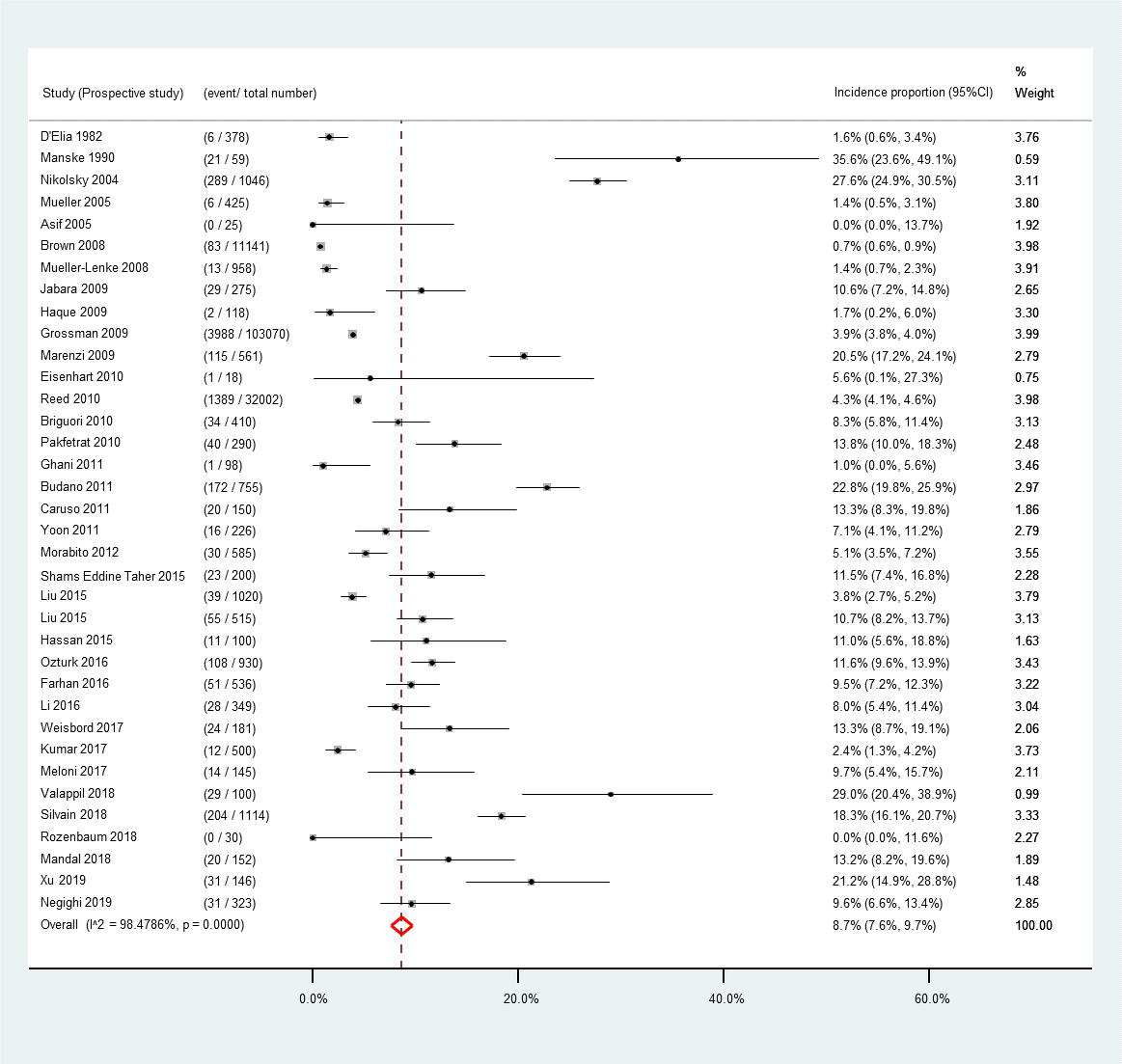
(B)


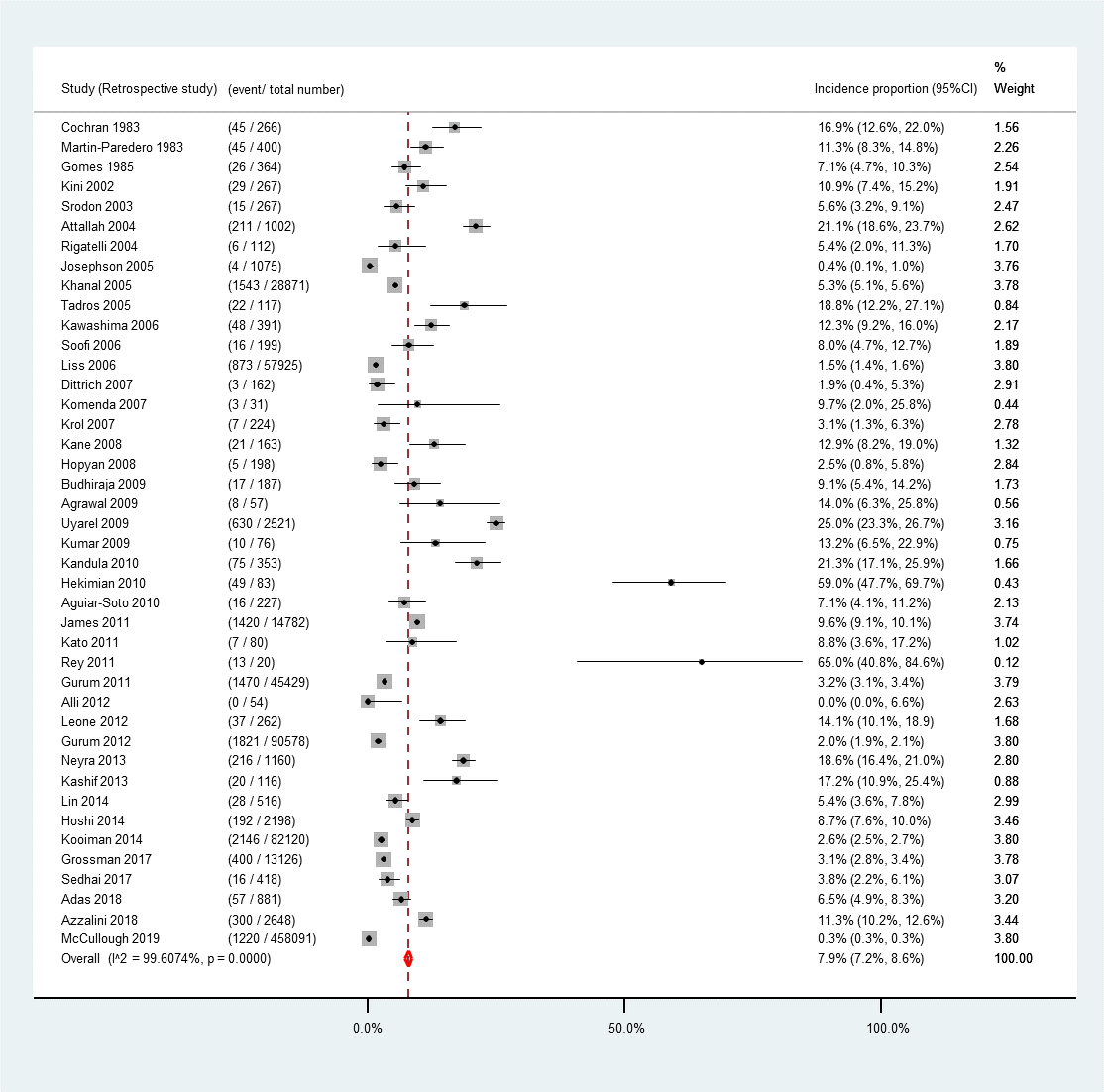
(C)

**Supplementary Figure 1.** Incidence proportion of contrast-induced nephropathy stratified by study designs. (A) RCT (placebo or control group); (B) Prospective study; (C) Retrospective study


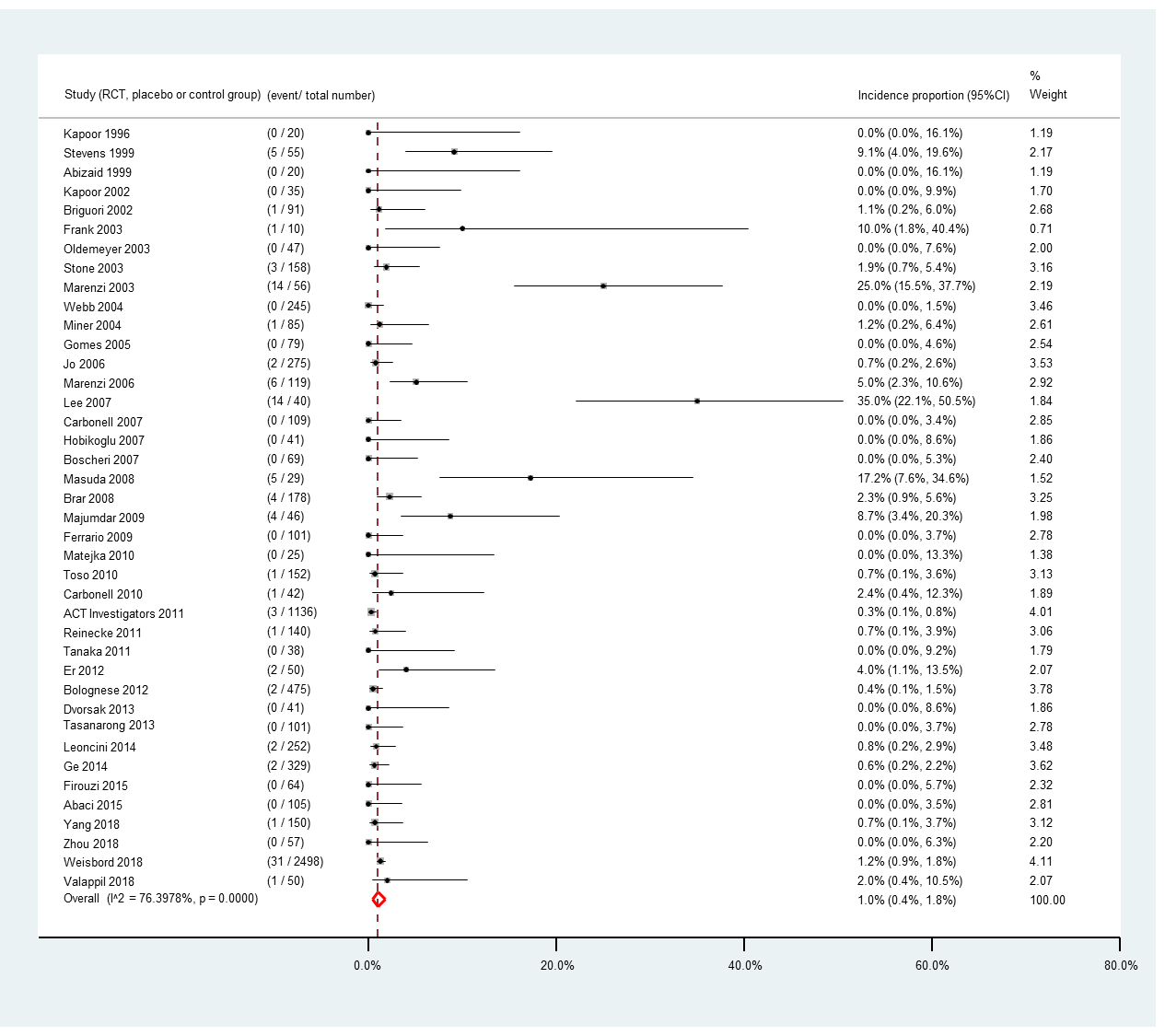
(A)


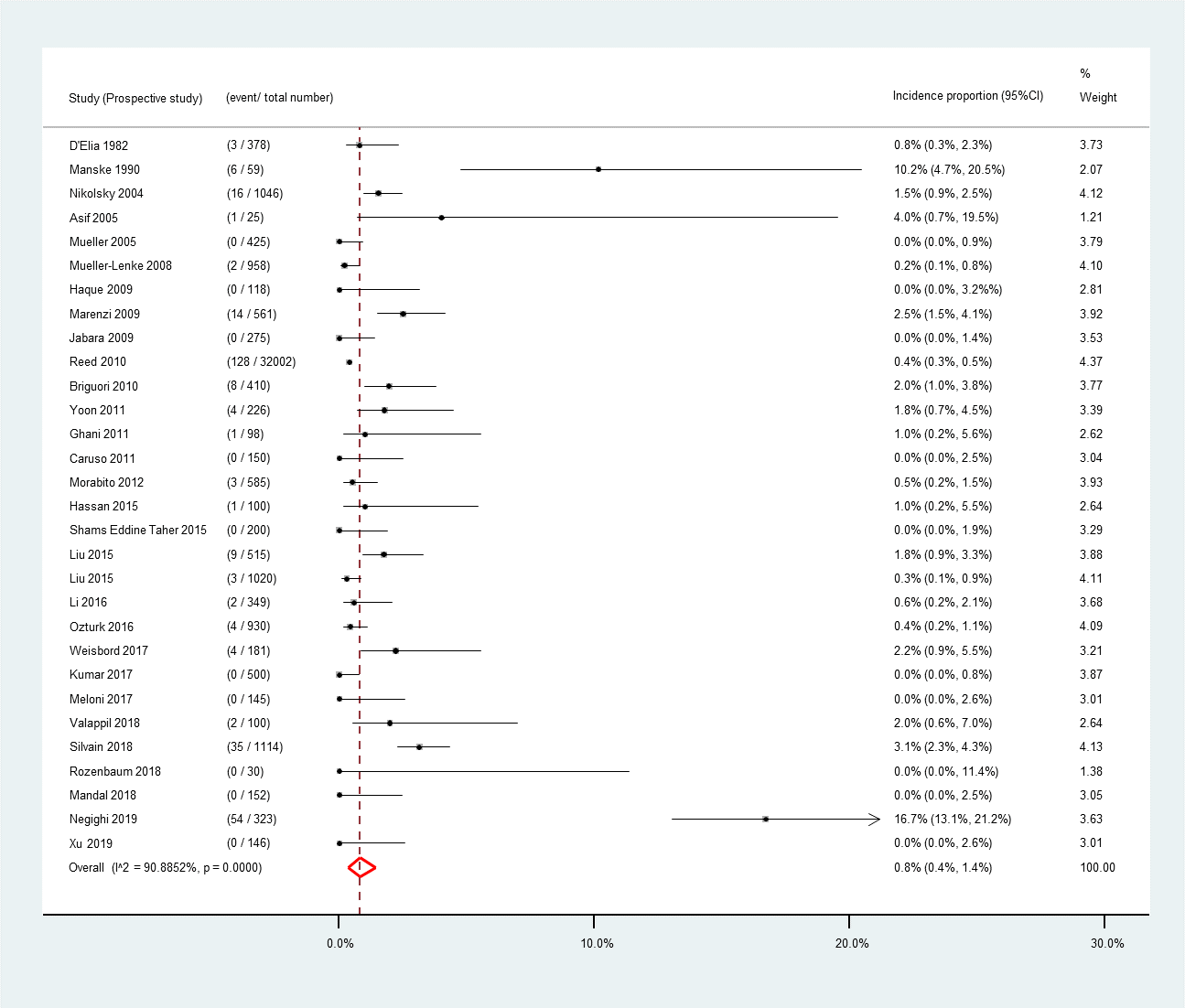
(B)


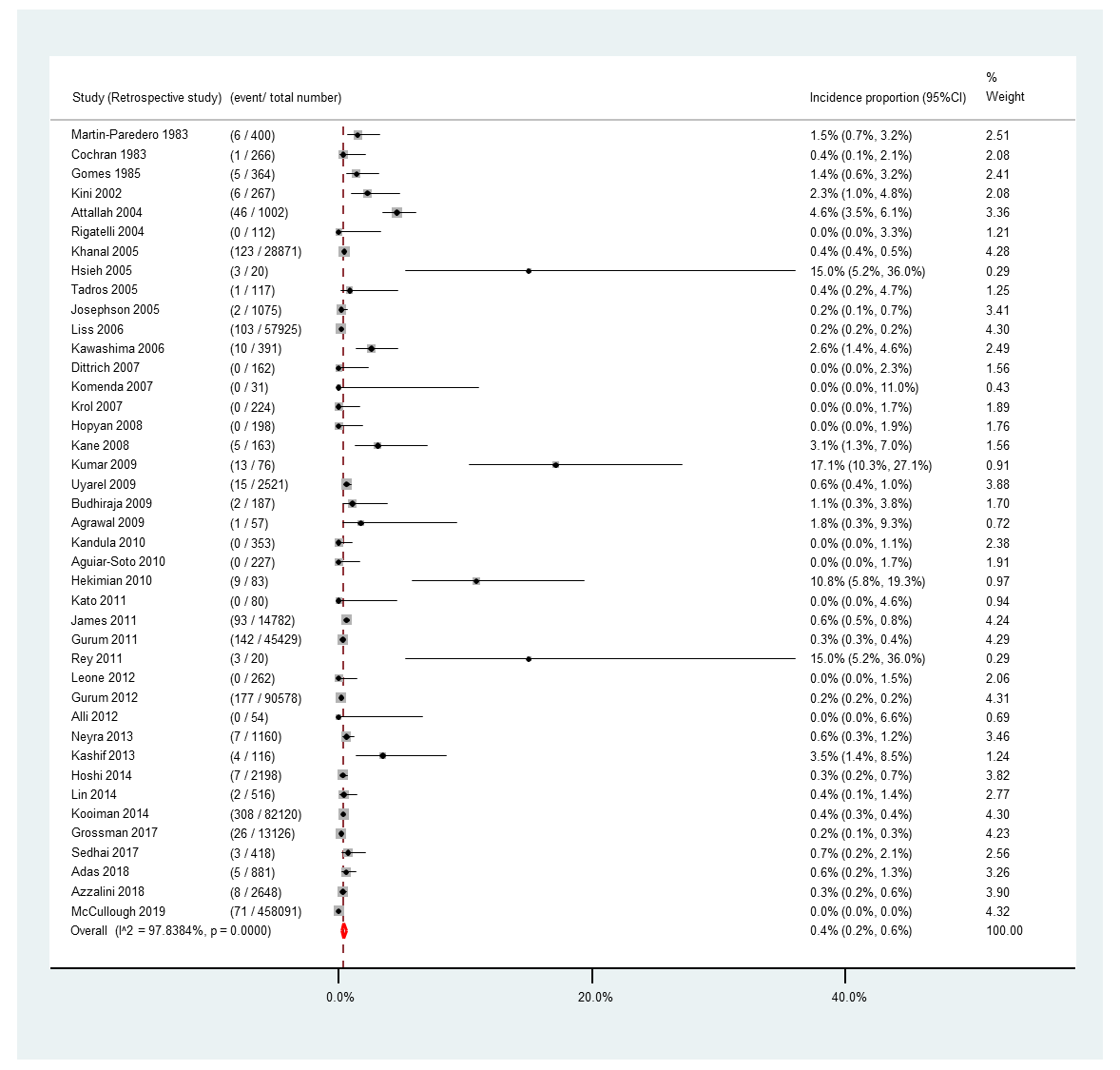
(C)

**Supplementary Figure 2.** Incidence proportion of renal replacement therapy stratified by study designs (A) RCT (placebo or control group); (B) Prospective study; (C) Retrospective study

(A)


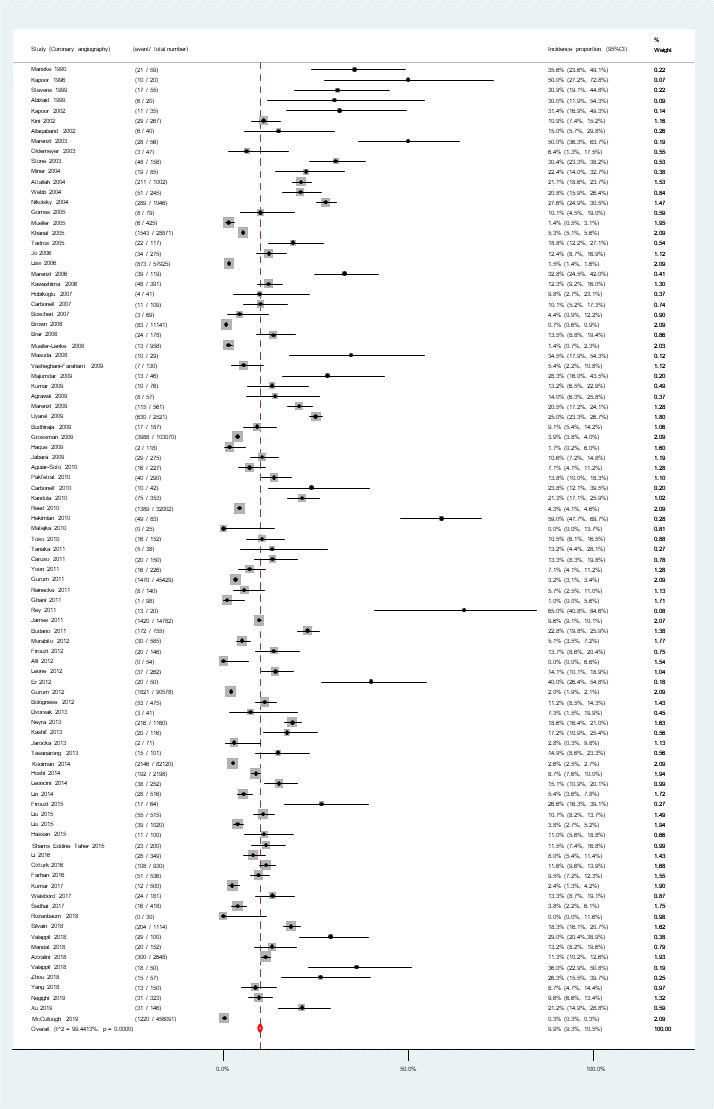


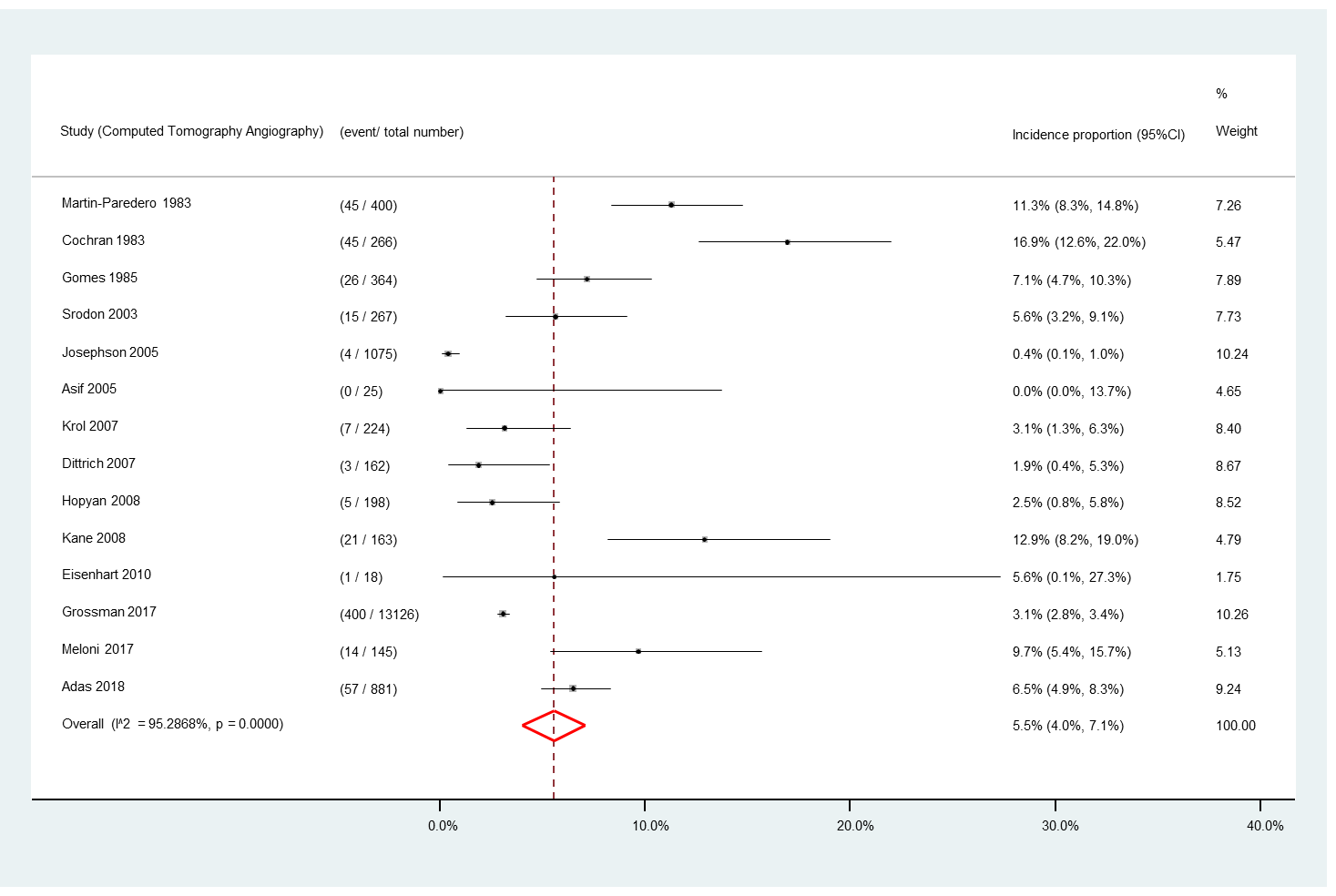
(B)


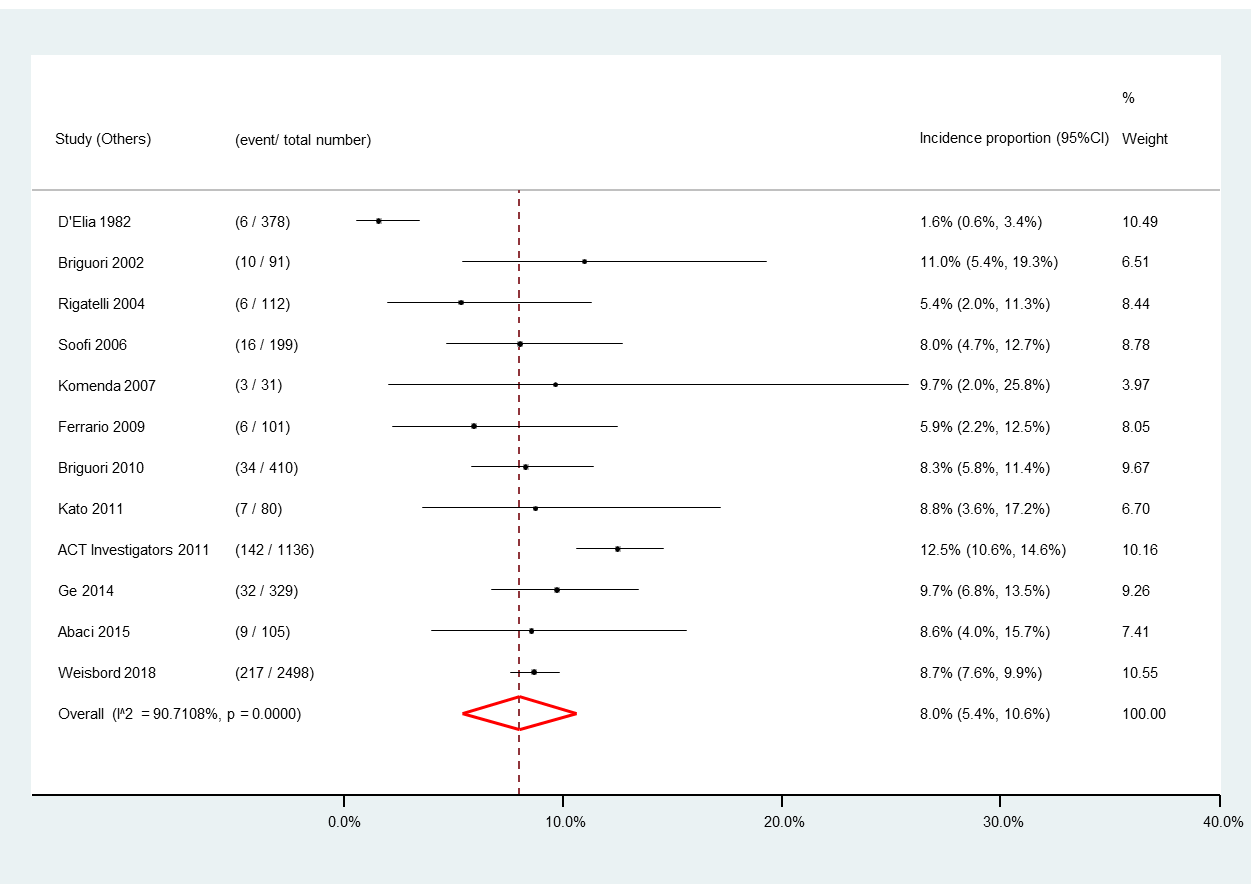
(C)

**Supplementary Figure 3.** Incidence proportion of contrast-induced nephropathy stratified by angiography procedure types. (A) Heart-related; (B) Non-Heart-related; (C) Others


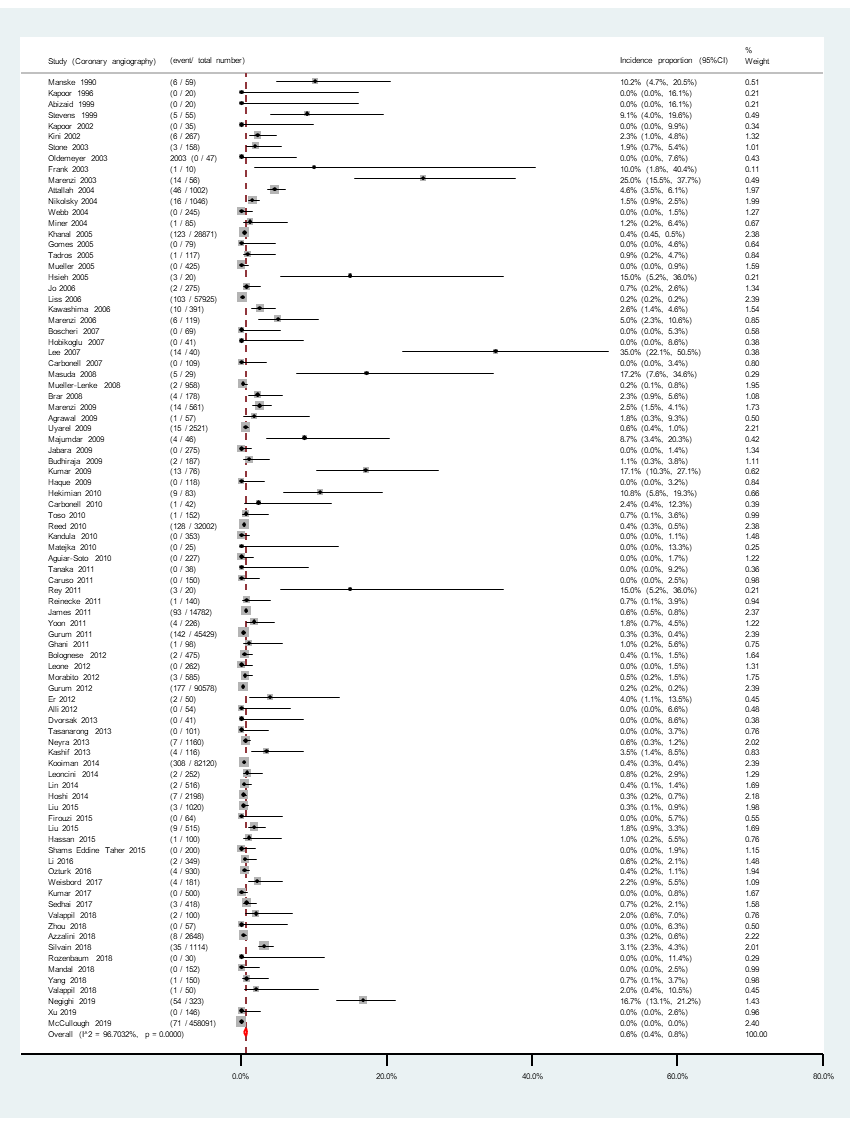
(A)


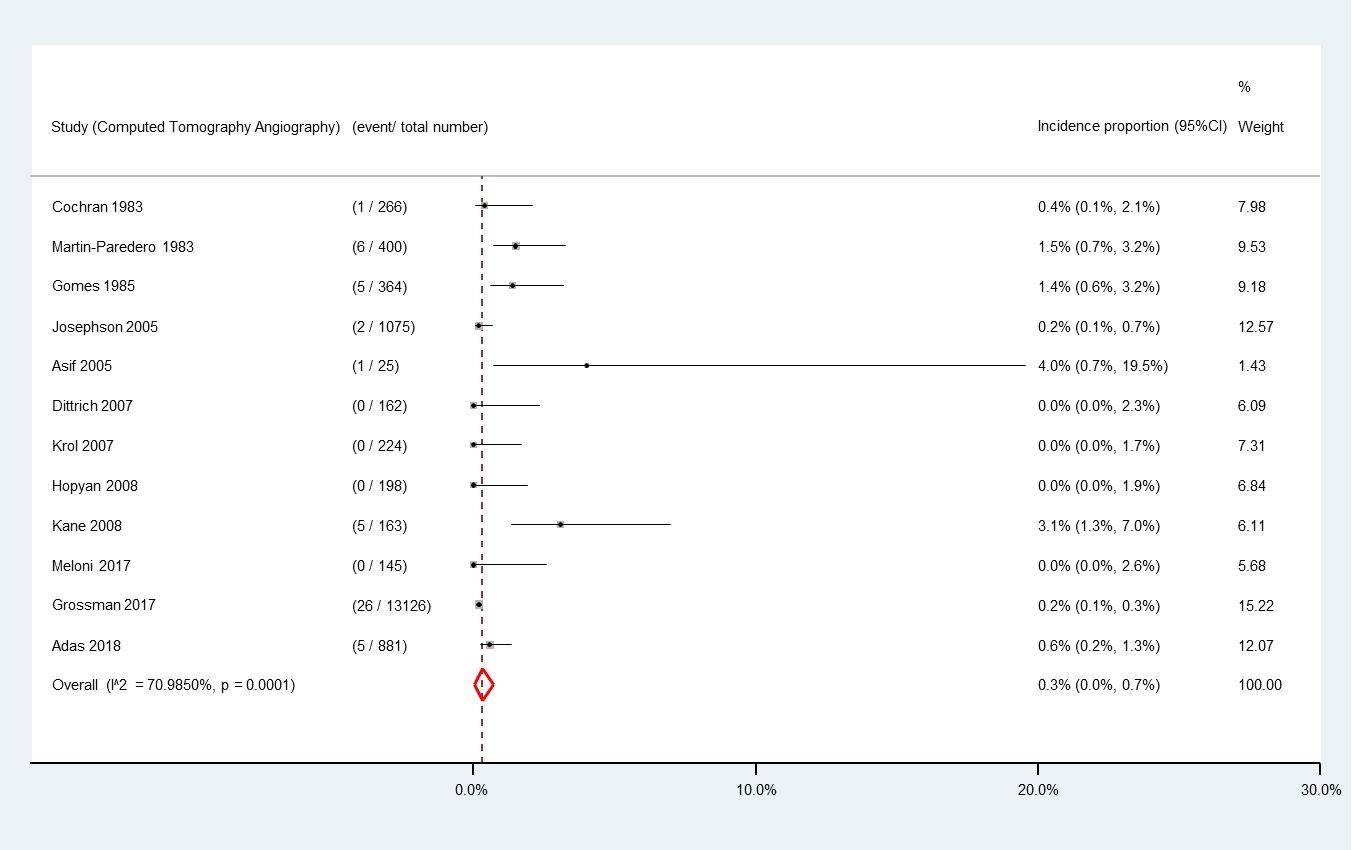
(B)


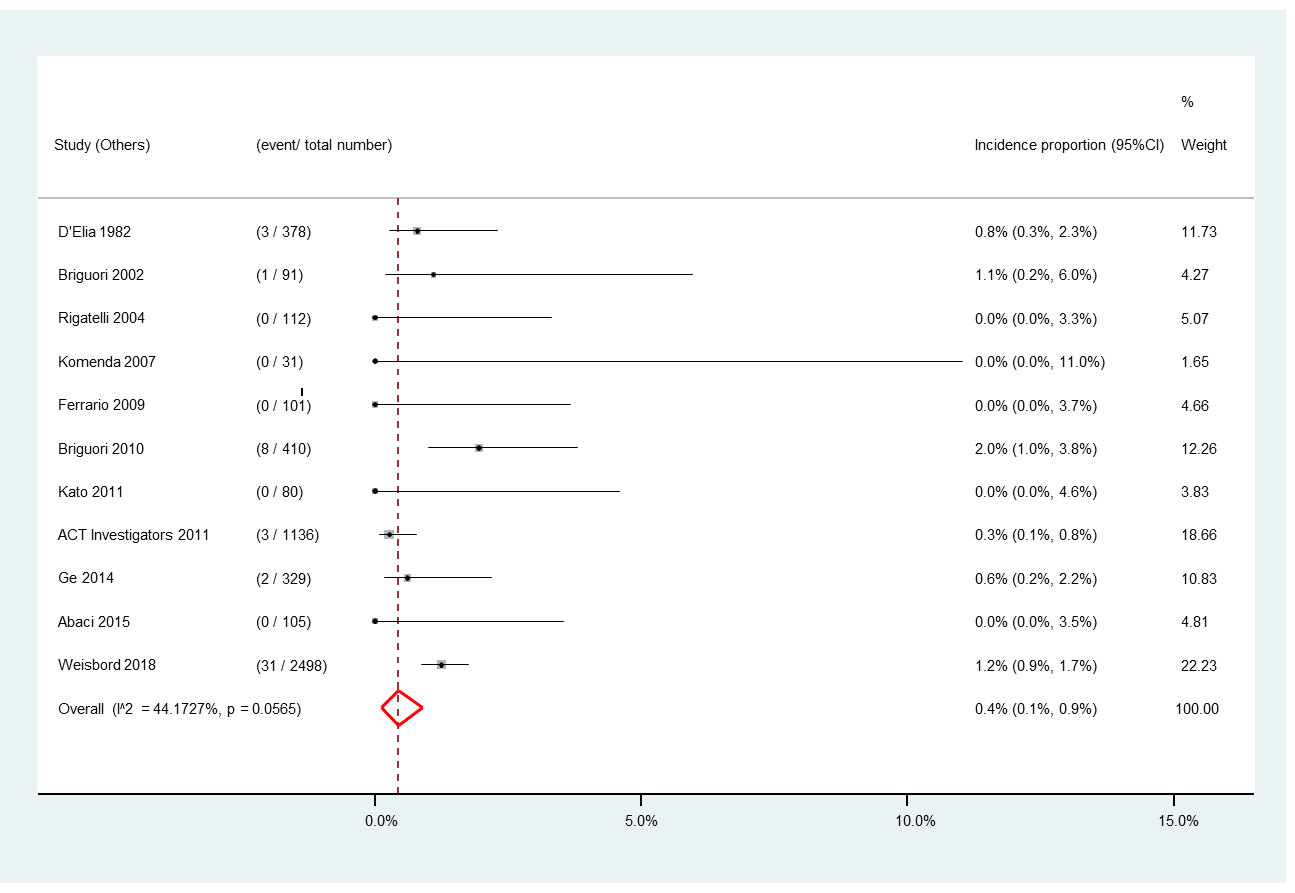
(C)

**Supplementary Figure 4.** Incidence proportion of renal replacement therapy stratified by angiography procedure types. (A) Heart-related; (B) Non-Heart-related; (C) Others

(A)


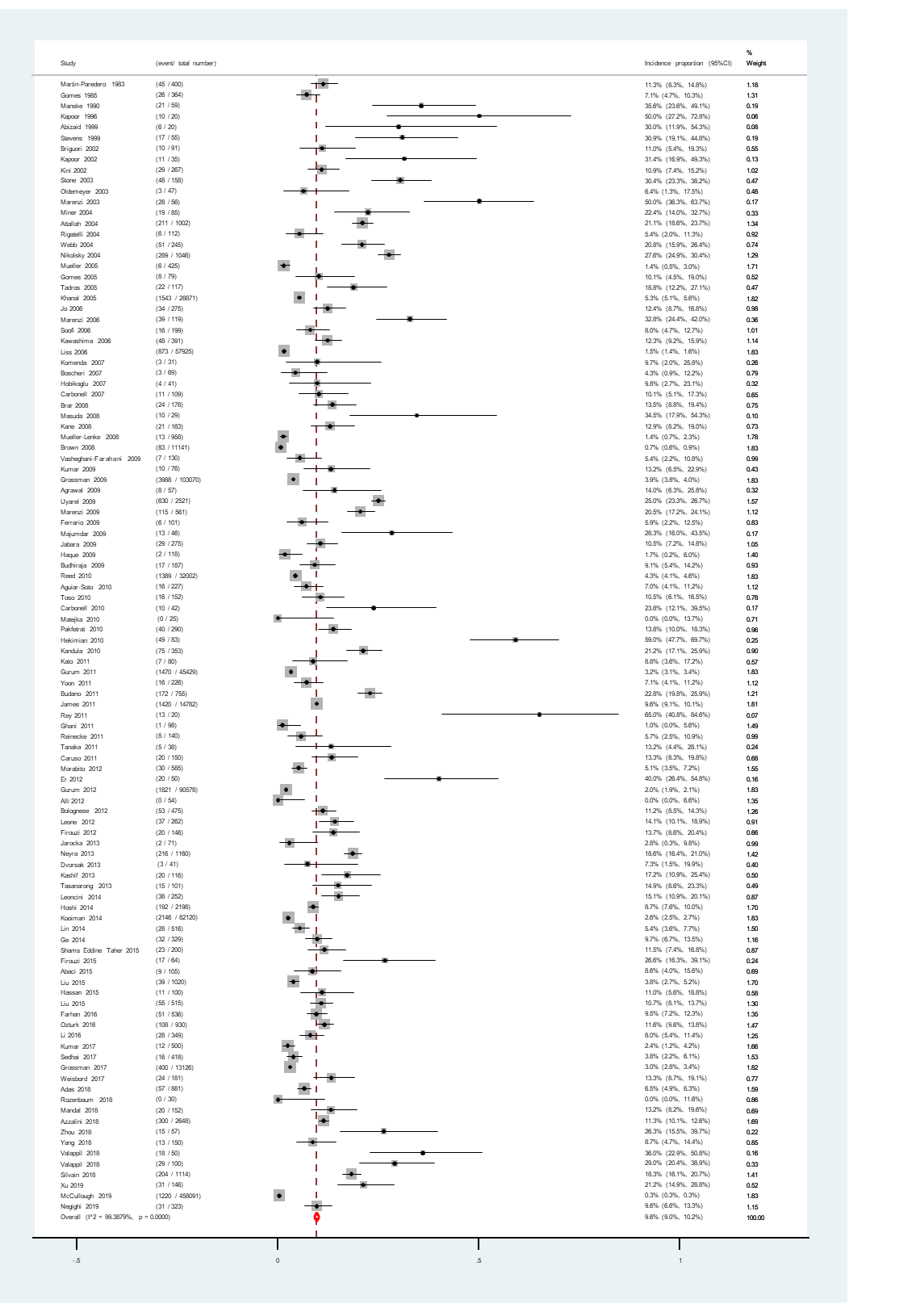


(B)


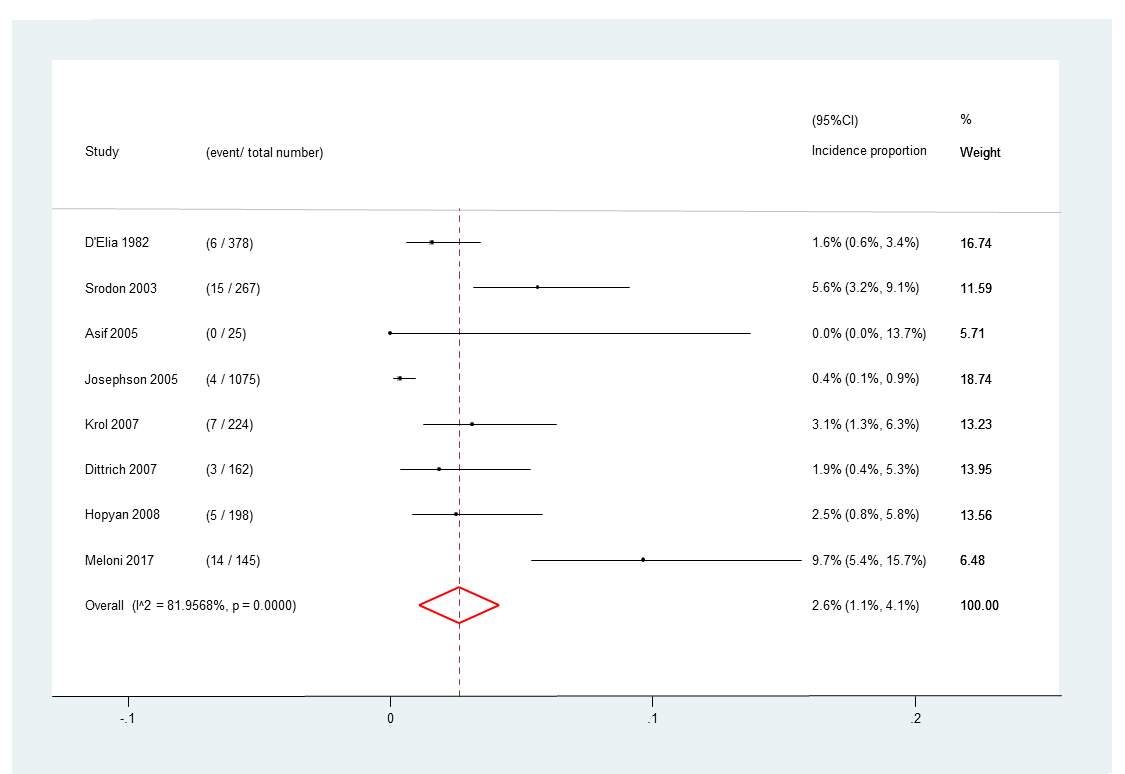


(C)


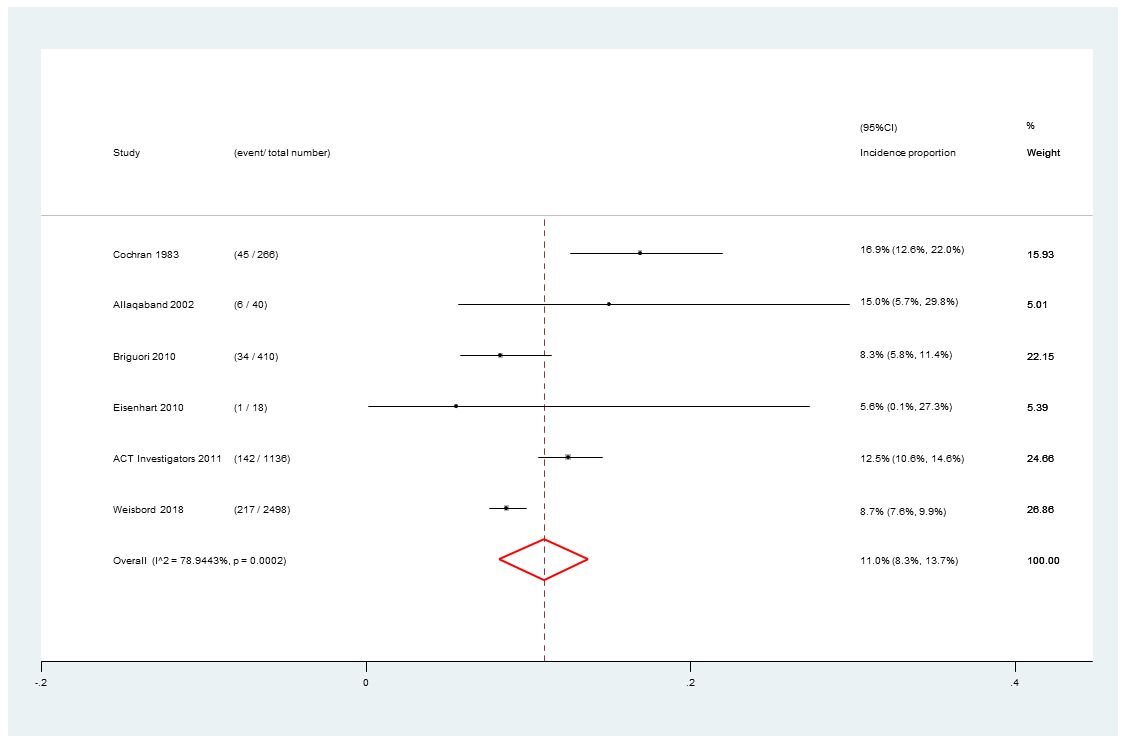


**Supplementary Figure 5.** Incidence proportion of contrast-induced nephropathy stratified by administration route. (A) Intra-arterial; (B) Intravenous; (C) Intra-arterial/intravenous

(A)


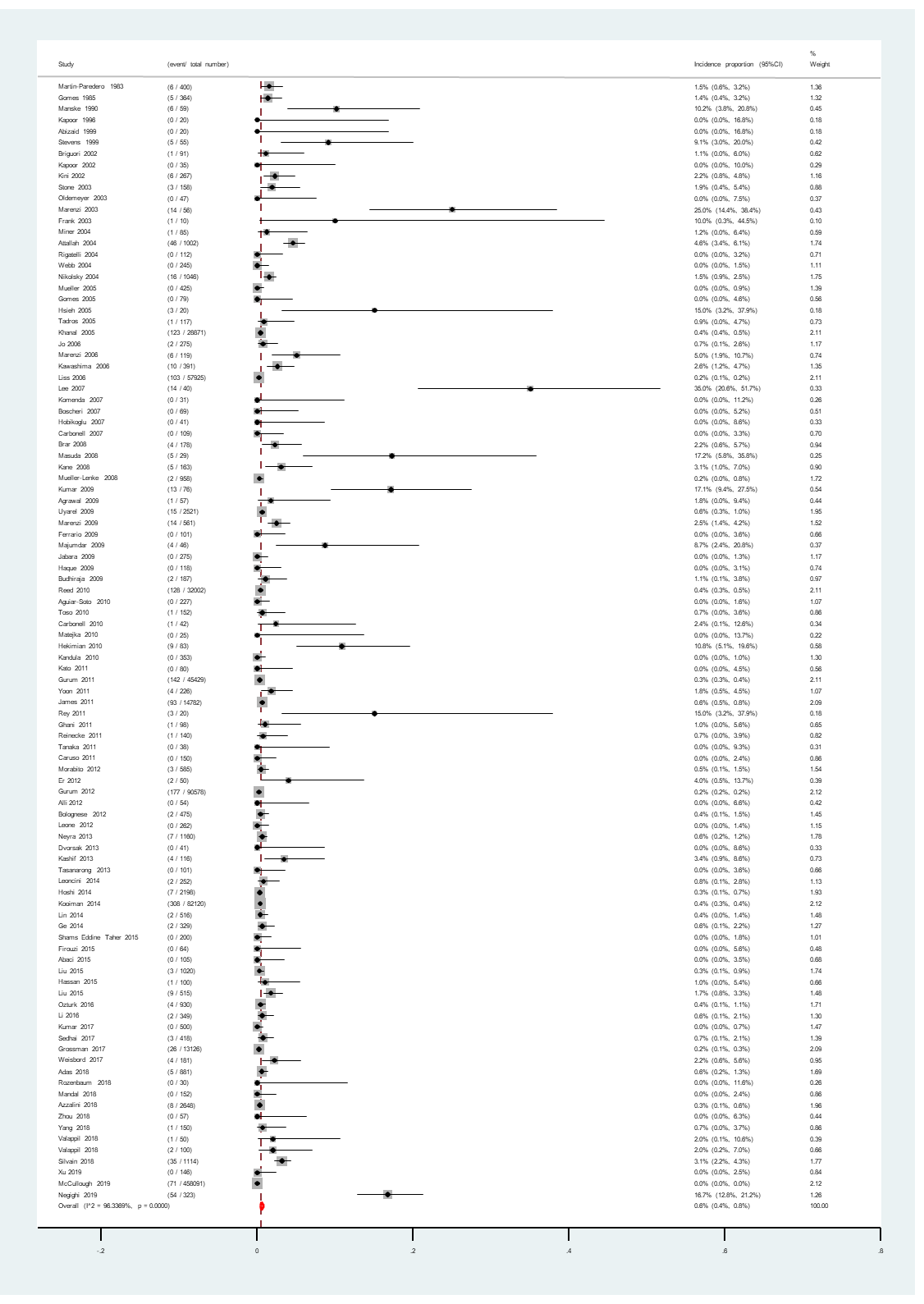


(B)


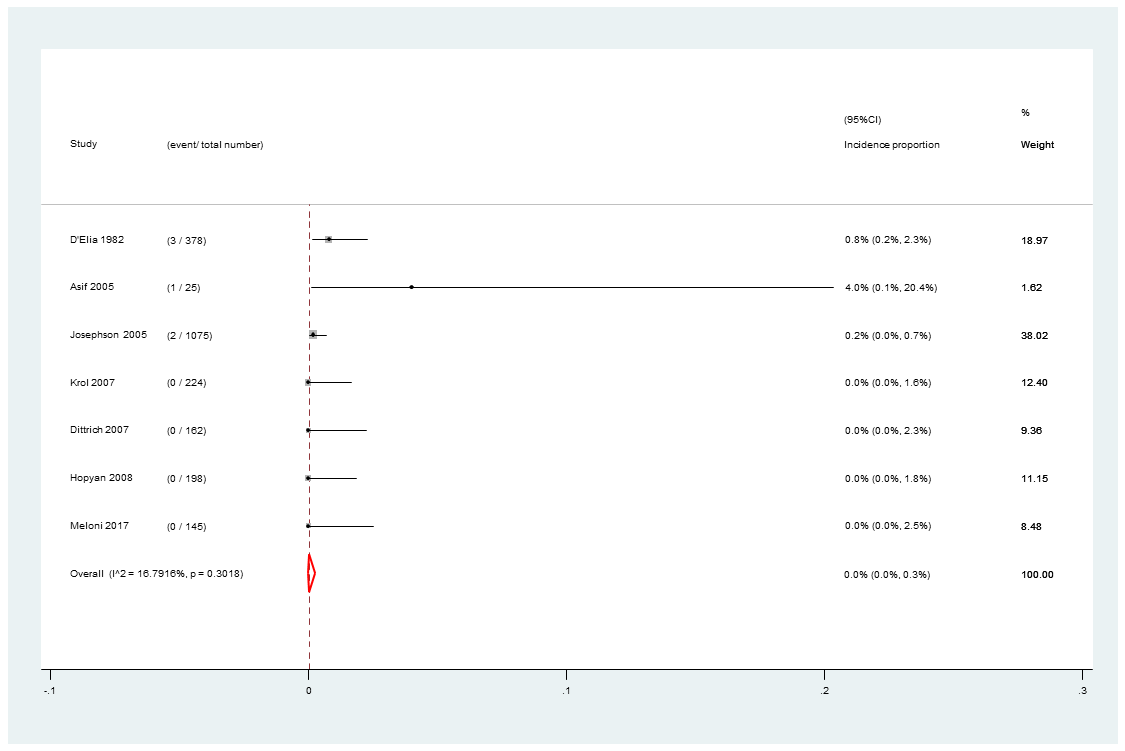


(C)


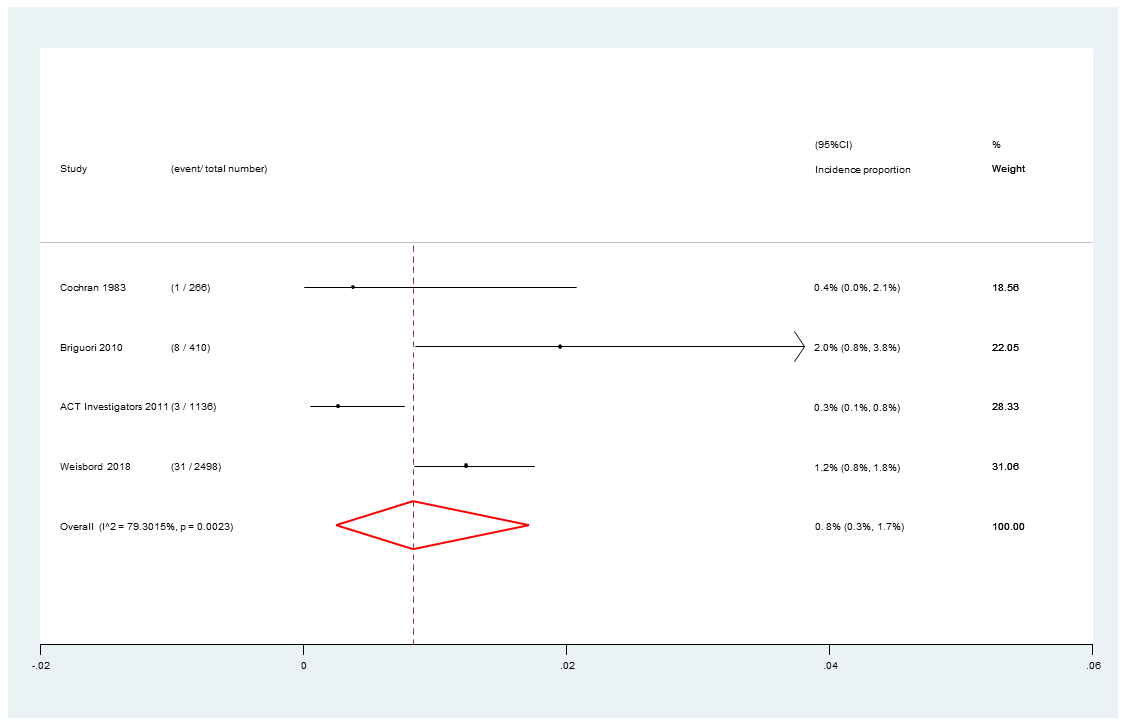


**Supplementary Figure 6.** Incidence proportion of renal replacement therapy stratified by administration route. (A) Intra-arterial; (B) Intravenous; (C) Intra-arterial/intravenous

(A)


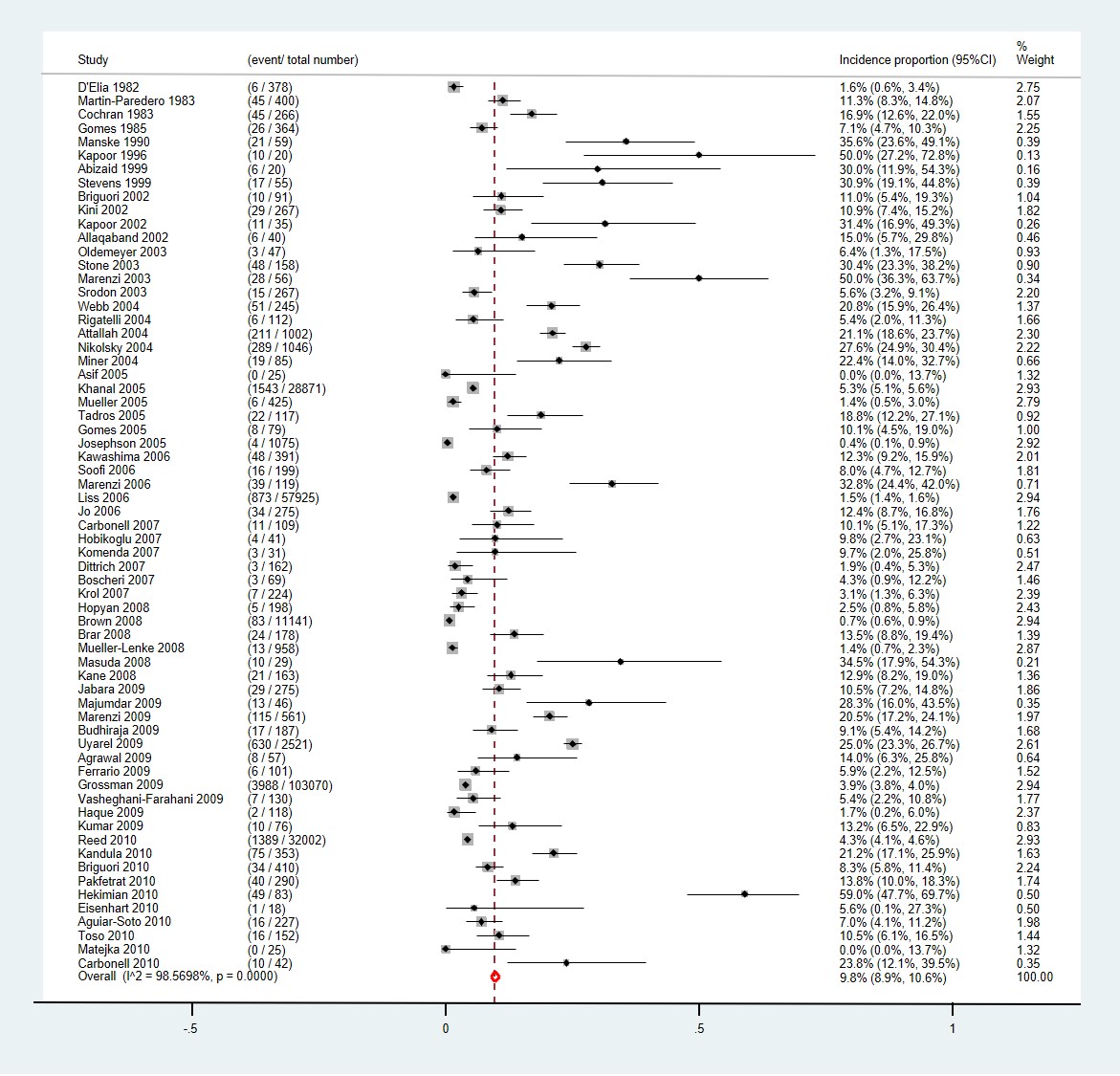


(B)


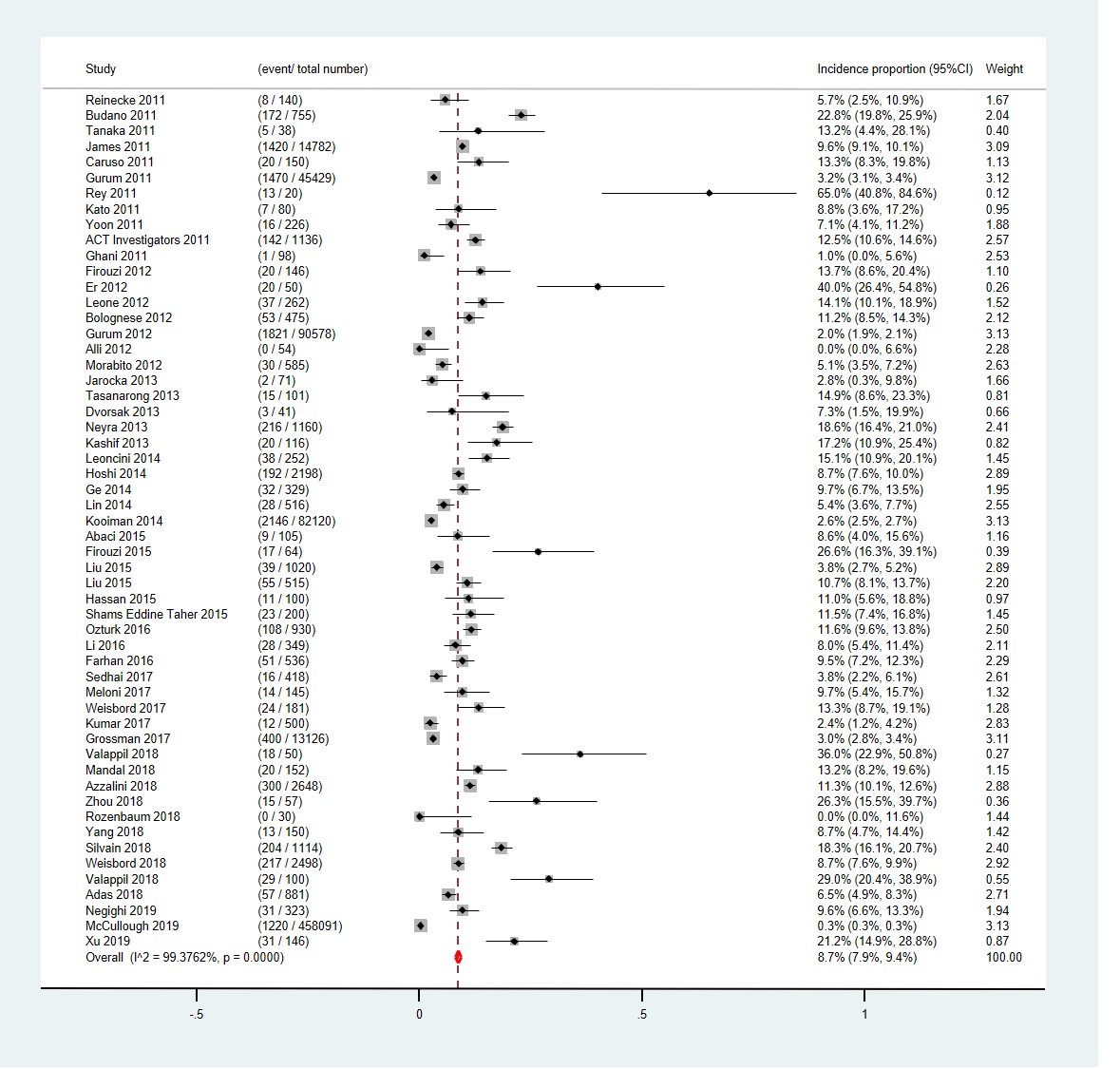


**Supplementary Figure 7.** Incidence proportion of contrast-induced nephropathy stratified by publication year. (A)Studies published before 2010; (B) Studies published after 2010

(A)


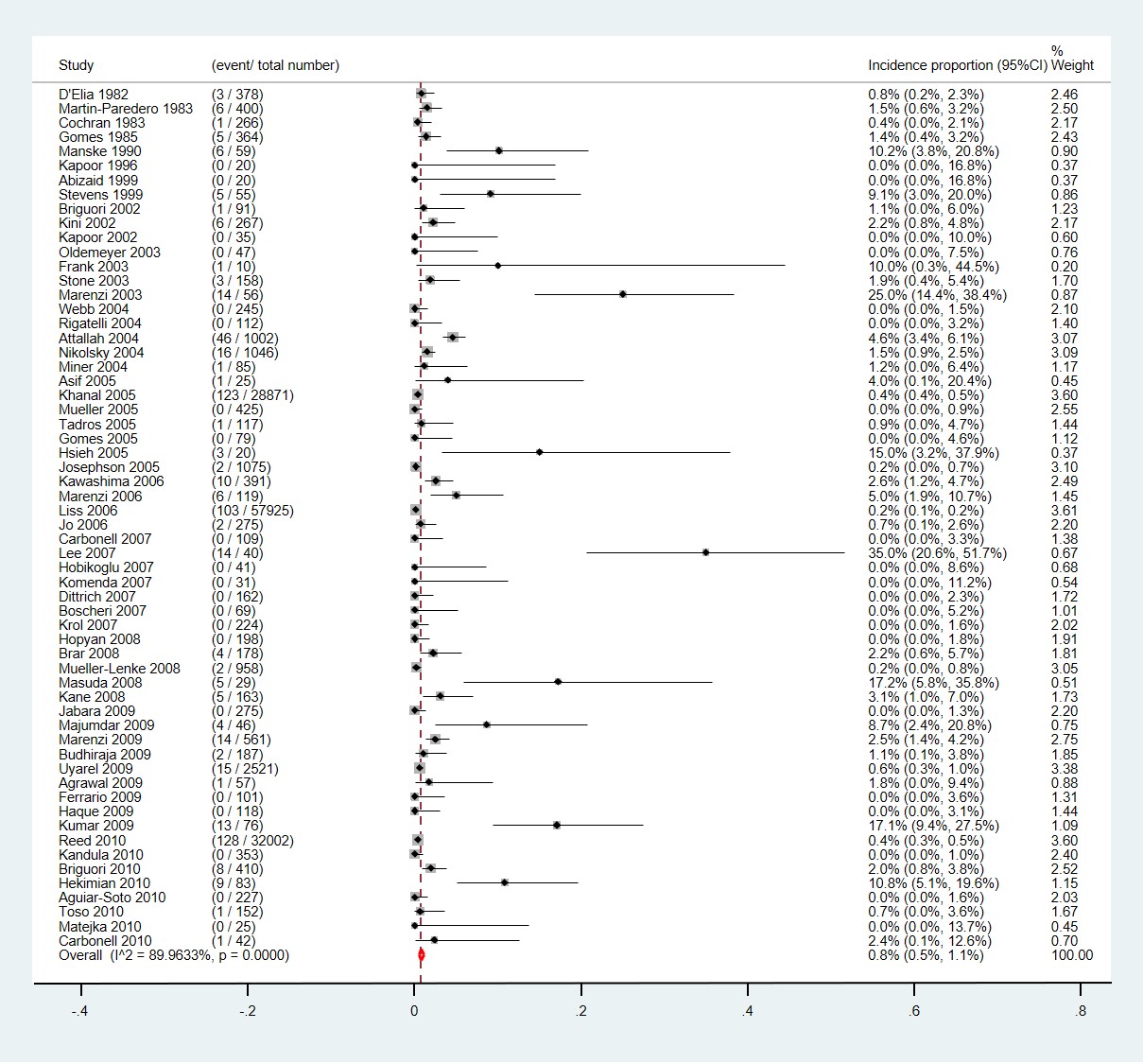


(B)


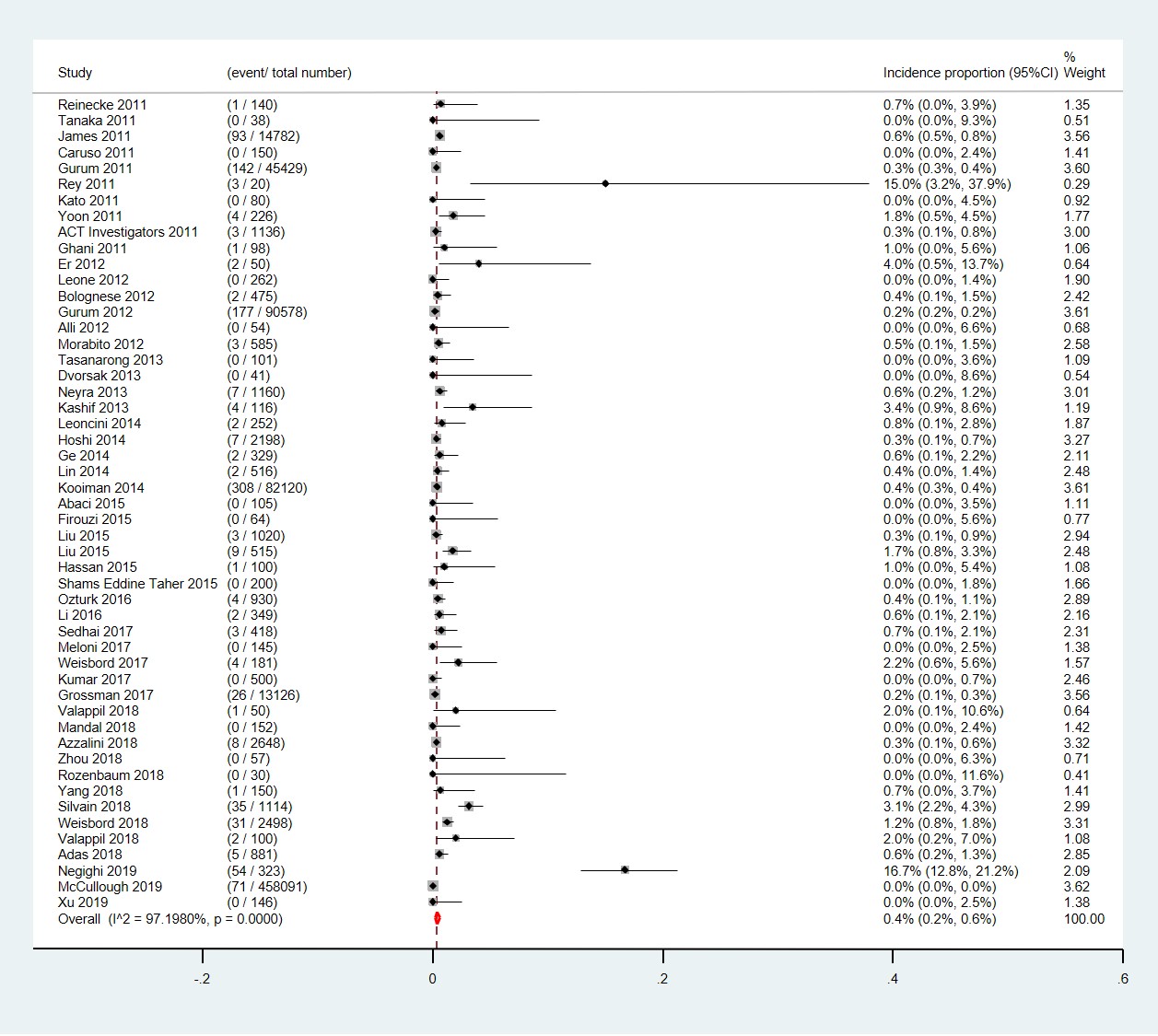


**Supplementary Figure 8.** Incidence proportion of renal replacement therapy stratified by publication year. (A)Studies published before 2010; (B) Studies published after 2010

(C)

(B)

(A)

**Supplementary Figure 9.** Doi plot analysis and Luis Furuya-Kanamori (LFK) index for detecting the risk of publication bias. Incidence proportion of contrast-induced nephropathy stratified by study designs. (A) RCT (placebo or control group); (B) Prospective study; (C) Retrospective study.

(C)

(B)

(A)

**Supplementary Figure 10.** Doi plot analysis and Luis Furuya-Kanamori (LFK) index for detecting the risk of publication bias. Incidence proportion of renal replacement therapy stratified by study designs. (A) RCT (placebo or control group); (B) Prospective study; (C) Retrospective study.


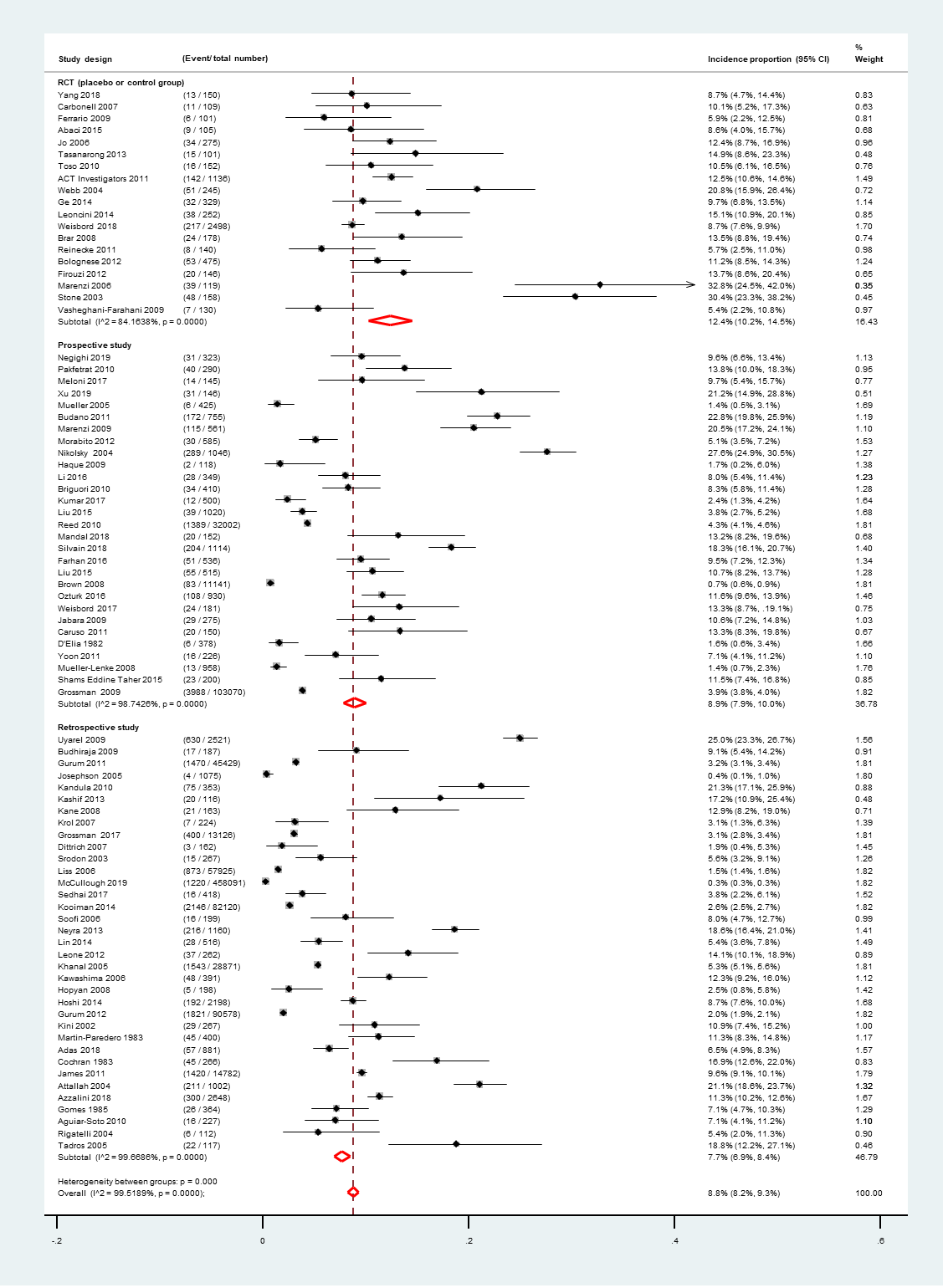


**Supplementary Figure 11.** Sensitivity analysis for incidence proportion of contrast-induced nephropathy stratified by study designs. (excluding the studies with sample size smaller than 100 participants)


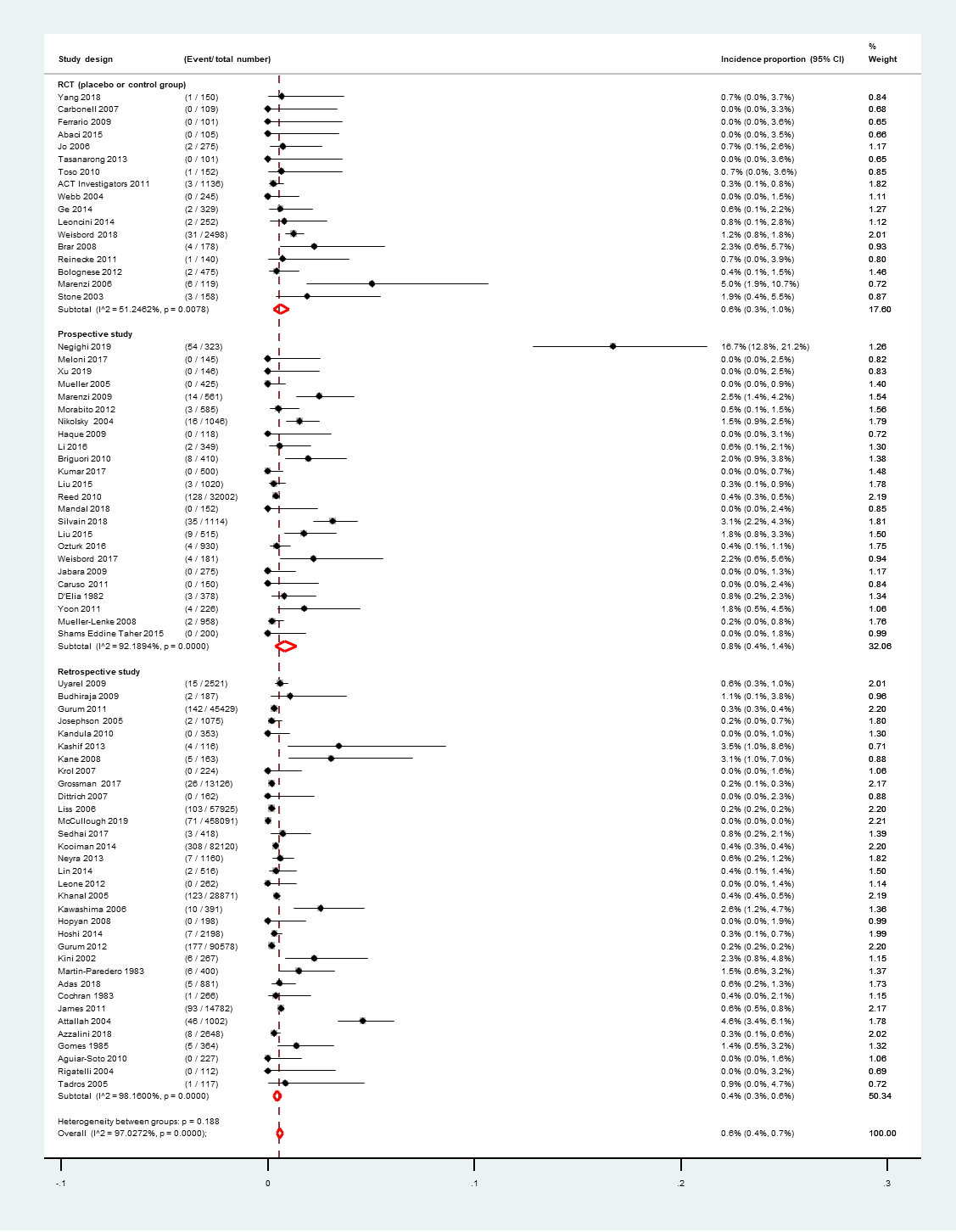


**Supplementary Figure 12.** Sensitivity analysis for incidence proportion of renal replacement therapy stratified by study designs. (excluding the studies with sample size smaller than 100 participants)

**Supplementary Table 1. Subgroup analysis for incidence proportion of contrast-induced nephropathy or renal replacement therapy**

| CIN | Study # | Incidence proportion | 95% CI | I^2^ | p-value | Test for heterogeneity between groups* |
| --- | --- | --- | --- | --- | --- | --- |
| Mean Age |  |  |  |  |  |  |
| <65 | 52 | 10.0% | (8.9%, 11.1%) | 99.41% | <0.0001 |  |
| 65+ | 58 | 10.9% | (10.0%, 11.8%) | 96.12% | <0.0001 | 0.4680 |
| Male (%) |  |  |  |  |  |  |
| <60% | 21 | 9.1% | (6.7%, 11.6%) | 95.22% | <0.0001 |  |
| >=60% | 89 | 10.1% | (9.4%, 10.7%) | 99.41% | <0.0001 | 0.7921 |
| Baseline creatinine (mg/dL) |  |  |  |  |  |  |
| <=1.2 | 41 | 11.7% | (10.4%, 13.0%) | 98.41% | <0.0001 |  |
| >1.2 | 49 | 14.6% | (12.5%, 16.6%) | 94.28% | <0.0001 | 0.0018 |
| eGFR< 60 ml/min/1.73m^2^ (%) |  |  |  |  |  |  |
| <100% | 21 | 11.3% | (8.6%, 13.9%) | 99.22% | <0.0001 |  |
| 100% | 26 | 11.2% | (9.1%, 13.3%) | 82.96% | <0.0001 | 0.5089 |
| Mean CM exposure (mL) |  |  |  |  |  |  |
| <=200 | 56 | 11.9% | (10.4%, 13.4%) | 90.18% | <0.0001 |  |
| >200 | 25 | 13.7% | (11.9%, 15.6%) | 98.46% | <0.0001 | 0.1814 |
| Days between CM administration and creatinine measurements |  |  |  |  |  |  |
| <=3 | 82 | 10.8% | (9.8%, 11.9%) | 97.80% | <0.0001 |  |
| >3 | 16 | 11.5% | (9.7%, 13.3%) | 89.54% | <0.0001 | 0.7815 |
| Follow-up time (days) |  |  |  |  |  |  |
| <30 | 26 | 12.9% | (9.4%, 16.4%) | 95.64% | <0.0001 |  |
| >=30 | 26 | 11.4% | (8.9%, 13.9%) | 98.77% | <0.0001 | 0.7533 |
| Representativeness score |  |  |  |  |  |  |
| 0 (low quality) | 58 | 14.3% | (12.2%, 16.4%) | 93.07% | <0.0001 |  |
| 1 (uncertain) | 9 | 16.0% | (9.7%, 22.2%) | 97.41% | <0.0001 |  |
| 2 (high quality) | 53 | 7.5% | (6.9%, 8.1%) | 99.57% | <0.0001 | <0.0001 |
|  |  |  |  |  |  |  |
| RRT |  |  |  |  |  |  |
| Mean Age |  |  |  |  |  |  |
| <65 | 46 | 0.54% | (0.28%, 0.87%) | 96.02% | <0.0001 |  |
| 65+ | 56 | 0.70% | (0.41%, 1.03%) | 88.16% | <0.0001 | 0.5675 |
| Male (%) |  |  |  |  |  |  |
| <60% | 20 | 0.95% | (0.24%, 1.98%) | 89.52% | <0.0001 |  |
| >=60% | 85 | 0.50% | (0.33%, 0.70%) | 96.20% | <0.0001 | 0.2516 |
| Baseline creatinine (mg/dL) |  |  |  |  |  |  |
| <=1.2 | 35 | 0.36% | (0.20%, 0.54%) | 73.00% | <0.0001 |  |
| >1.2 | 48 | 2.07% | (1.17%, 3.16%) | 91.19% | <0.0001 | <0.0001 |
| eGFR< 60 ml/min/1.73m^2^ (%) |  |  |  |  |  |  |
| <100% | 19 | 0.21% | (0.08%, 0.38%) | 79.58% | <0.0001 |  |
| 100% | 24 | 1.69% | (0.68%, 3.03%) | 85.28% | <0.0001 | <0.0001 |
| Mean CM exposure (mL) |  |  |  |  |  |  |
| <=200 | 53 | 0.85% | (0.04%, 1.43%) | 84.75% | <0.0001 |  |
| >200 | 22 | 0.65% | (0.35%, 1.02%) | 88.76% | <0.0001 | 0.2438 |
| Days between CM administration and creatinine measurements |  |  |  |  |  |  |
| <=3 | 73 | 0.56% | (0.34%, 0.80%) | 86.55% | <0.0001 |  |
| >3 | 17 | 0.75% | (0.28%, 1.37%) | 84.84% | <0.0001 | 0.5170 |
| Follow-up time (days) |  |  |  |  |  |  |
| <30 | 26 | 0.57% | (0.14%, 1.20%) | 72.39% | <0.0001 |  |
| >=30 | 28 | 0.51% | (0.20%, 0.92%) | 89.45% | <0.0001 | 0.9507 |
| Representativeness score |  |  |  |  |  |  |
| 0 (low quality) | 58 | 1.82% | (1.08%, 2.71%) | 84.75% | <0.0001 |  |
| 1 (uncertain) | 7 | 1.55% | (0.30%, 3.45%) | 73.58% | <0.0001 |  |
| 2 (high quality) | 46 | 0.25% | (0.14%, 0.39%) | 97.42% | <0.0001 | <0.0001 |

*: p-value of test for heterogeneity between groups

**Supplementary Table 2. key information and quality assessment of each included study**

| Study year | Study design | Country | Intervention | Mean age | Male | Baseline creatinine (mg/dL) (mean±SD) | | | eGFR< 60 ml/min/1.73m^2^ | Mean CM exposure (mL) | Representativeness  of the population^$^ | Clear case definition^$^ | Response rate^$^ | Sufficient follow‐up time^$^ |
| --- | --- | --- | --- | --- | --- | --- | --- | --- | --- | --- | --- | --- | --- | --- |
| ACT Investigators, 2011 | RCT | BRA | coronary or peripheral arterial diagnostic intravascular angiography or percutaneous intervention | 68.1 | 60.7% | 1.20 | ± | 0.50 | 40.0% | - | 2 | 2 | 2 | 2 |
| Abaci et al., 2015 | RCT | TUR | coronary angiography or peripheral angiography | 67.7 | 73.4% | 1.40 | ± | 0.50 | 100.0% | 117.70 | 0 | 2 | 2 | 2 |
| Abizaid et al., 1999 | RCT | USA | coronary angioplasty | 75.0 | 70.0% | 2.30 | ± | 0.80 | - | 182.00 | 0 | 2 | 2 | 0 |
| Agrawal et al., 2009 | Retrospective | USA | cardiac catheterization | 58.2 | 73.7% | 1.70 | ± | 0.80 | 66.7% | - | 0 | 2 | 2 | 0 |
| Aguiar-Soto et al., 2010 | Retrospective | GBR | percutaneous coronary intervention | 64.0 | 81.9% | 72.00 | ± | 20.00 | 28.0% | 260.00 | 2 | 2 | 2 | 0 |
| Adas et al., 2018 | Retrospective | USA | peripheral vascular interventions | - | - | - | | | - | - | 2 | 2 | 2 | 2 |
| Allaqaband et al., 2002 | RCT | USA | cardiovascular procedures | 71.0 | 60.0% | 2.03 | ± | 0.79 | - | 154.00 | 0 | 2 | 2 | 0 |
| Alli et al., 2012 | Retrospective | USA | percutaneous coronary intervention (Tandem Heart assisted) | 72.0 | 78.0% | 1.60 | ± | 0.30 | - | - | 0 | 0 | 2 | 1 |
| Asif et al., 2005 | Prospective | USA | venography | 48.9 | 56.0% | - | | | 100.0% | - | 0 | 2 | 2 | 2 |
| Attallah et al., 2004 | Retrospective | USA | cardiac catheterization | 61.8 | 46.8% | 2.00 | ± | -- | - | - | 0 | 2 | 2 | 0 |
| Azzalini et al., 2018 | Retrospective | ITA | percutaneous coronary intervention | 68.1 | 81.0% | - | | | 34.4% | - | 2 | 2 | 0 | 0 |
| Bolognese et al., 2012 | RCT | ITA | percutaneous coronary intervention | 65.5 | 77.0% | 1.06 | ± | 0.45 | - | 157.00 | 2 | 2 | 2 | 2 |
| Boscheri et al., 2007 | RCT | DEU | coronary angiograpy/procedures | 71.0 | 71.0% | 1.73 | ± | 0.40 | 100.0% | 112.00 | 0 | 2 | 2 | 0 |
| Brar et al., 2008 | RCT | USA | coronary angiography | 71.0 | 64.8% | - | | | 100.0% | 137.00 | 0 | 2 | 2 | 2 |
| Briguori et al., 2002 | RCT | ITA | coronary and/or peripheral angiography and/or angioplasty | 64.0 | 89.0% | 1.54 | ± | 0.36 | - | 200.00 | 0 | 2 | 2 | 0 |
| Briguori et al., 2010 | Prospective | ITA | coronary and/or peripheral angiography and/or angioplasty | 70.0 | 84.0% | 1.64 | ± | -- | 100.0% | 165.00 | 0 | 2 | 2 | 2 |
| Brown et al., 2008 | Prospective | USA | percutaneous coronary intervention | - | - | - | | | - | - | 2 | 1 | 2 | 1 |
| Budano et al., 2011 | Prospective | ITA | coronary angiography | 66.0 | 72.8% | 1.11 | ± | 0.96 | 20.9% | 230.00 | 2 | 2 | 2 | 1 |
| Budhiraja et al., 2009 | Retrospective | USA | coronary angiogram | - | 100.0% | - | | | - | - | 0 | 2 | 2 | 2 |
| Carbonell et al., 2007 | RCT | ESP | coronary angiography | 60.6 | 72.5% | 0.96 | ± | 0.17 | - | 183.00 | 2 | 2 | 2 | 0 |
| Carbonell et al., 2010 | RCT | ESP | coronary angiography | - | 81.0% | 1.87 | ± | 0.70 | - | 184.66 | 0 | 2 | 2 | 1 |
| Caruso et al., 2011 | Prospective | ITA | percutaneous coronary intervention | 65.9 | 72.7% | 0.99 | ± | 0.36 | 16.0% | 113.60 | 2 | 2 | 2 | 2 |
| Cochran et al., 1983 | Retrospective | USA | renal angiography | 47.8 | 56.8% | - | | | - | - | 2 | 1 | 2 | 0 |
| D'Elia et al., 1982 | Prospective | USA | nonrenal angiography | 59.0 | 51.6% | - | | | - | - | 2 | 2 | 2 | 0 |
| Dittrich et al., 2007 | Retrospective | DEU | CT perfusion and CT angiography | 66.0 | 64.0% | 1.10 | ± | 0.30 | - | 140.00 | 2 | 2 | 2 | 0 |
| Dvorsak et al., 2013 | RCT | SVN | coronary angiography or angioplasty | 70.7 | 68.3% | 1.51 | ± | 0.35 | - | 130.60 | 0 | 2 | 2 | 0 |
| Eisenhart et al., 2010 | Prospective | USA | low volume iodinated contrast administration for fistulography and angioplasty | 69.5 | 56.0% | 3.70 | ± | 2.11 | 100.0% | - | 0 | 2 | 0 | 0 |
| Er et al., 2012 | RCT | DEU | coronary angiography | 72.7 | 74.0% | 1.62 | ± | -- | 100.0% | 103.00 | 0 | 2 | 2 | 2 |
| Farhan et al., 2016 | Prospective | AUT | percutaneous coronary intervention | 62.7 | 68.1% | 1.07 | ± | -- | - | 255.42 | 2 | 2 | 1 | 2 |
| Ferrario et al., 2009 | RCT | ITA | diagnostic and interventional angiography | 75.0 | 62.0% | - | | | - | 168.00 | 0 | 2 | 2 | 0 |
| Firouzi et al., 2012 | RCT | IRN | coronary angioplasty | 57.9 | 69.2% | 1.21 | ± | 0.24 | - | 325.34 | 2 | 2 | 2 | 0 |
| Firouzi et al., 2015 | RCT | IRN | coronary angiography and intervention | 57.9 | 66.1% | 1.21 | ± | 0.24 | - | 324.34 | 0 | 2 | 1 | 0 |
| Frank et al., 2003 | RCT | DEU | coronary angiography | 57.6 | 90.0% | 4.20 | ± | 1.10 | - | - | 0 | 0 | 1 | 2 |
| Ge et al., 2014 | RCT | CHN | coronary or peripheral intervention | 63.2 | 66.3% | 1.15 | ± | 0.29 | - | 154.65 | 0 | 2 | 2 | 2 |
| Ghani et al., 2011 | Prospective | KWT | coronary angiography | 60.7 | 53.1% | 4.65 | ± | 0.90 | - |  | 0 | 2 | 2 | 0 |
| Gomes et al., 1985 | Retrospective | USA | major arteriography | 61.8 | 58.5% | - | | | - | 216.65 | 1 | 2 | 2 | 0 |
| Gomes et al., 2005 | RCT | BRA | coronary angiography or percutaneous coronary intervention | 66.5 | 57.0% | 1.26 | ± | 0.35 | - | 102.80 | 1 | 2 | 2 | 0 |
| Grossman et al., 2017 | Retrospective | USA | percutaneous vascular intervention | 69.0 | 54.3% | - | | | - | - | 2 | 2 | 1 | 1 |
| Grossman et al., 2009 | Prospective | USA | percutaneous coronary intervention | 63.8 | 65.7% | 1.18 | ± | -- | - | - | 1 | 2 | 2 | 0 |
| Gurum et al., 2011 | Retrospective BMC2 registry | USA | percutaneous coronary intervention | - | 65.9% | - | | | - | - | 2 | 2 | 2 | 0 |
| Gurum et al., 2012 | Retrospective BMC2 registry | USA | percutaneous coronary intervention | - | 62.6% | - | | | 71.0% | - | 2 | 2 | 2 | 0 |
| Haque et al., 2009 | Prospective | BGD | coronary angiography | - | 69.5% | - | | | - | - | 2 | 2 | 2 | 0 |
| Hassan et al., 2015 | Prospective | EGY | percutaneous coronary intervention | 56.0 | 76.0% | 0.95 | ± | 0.31 | - | 160.00 | 2 | 2 | 2 | 2 |
| Hekimian et al., 2010 | Retrospective | FRA | coronary angiography | 52.0 | 68.0% | 1.49 | ± | 1.16 | - | - | 1 | 1 | 2 | 1 |
| Hobikoglu et al., 2007 | RCT | TUR | coronary angiography | 62.0 | 71.6% | 1.57 | ± | 0.16 | - | 118.00 | 1 | 2 | 2 | 0 |
| Hopyan et al., 2008 | Retrospective | CAN | contrast-enhanced CT angiography (CTA) and CT perfusion (CTP) | 65.4 | 50.0% | - | | | - | - | 0 | 2 | 2 | 0 |
| Hoshi et al., 2014 | Retrospective | JPN | percutaneous coronary intervention | 70.0 | 76.0% | 0.91 | ± | 0.40 | - | 174.00 | 2 | 2 | 2 | 2 |
| Hsieh et al., 2005 | Retrospective | TWN | cardiovascular catheterizations | 66.0 | 80.0% | 3.50 | ± | 0.20 | - | - | 0 | 1 | 2 | 2 |
| Jabara et al., 2009 | prospective | USA | percutaneous coronary intervention | 62.0 | 81.0% | 1.12 | ± | 0.30 | 24.3% | 238.00 | 2 | 2 | 0 | 0 |
| James et al., 2011 | Retrospective | CAN | coronary angiography | 66.0 | 70.0% | 1.07 | ± | -- | 24.0% | - | 2 | 2 | 2 | 2 |
| Jarocka et al., 2013 | RCT | POL | percutaneous coronary intervention | 63.3 | - | 0.97 | ± | 0.19 | - | 152.39 | 1 | 2 | 2 | 1 |
| Jo et al., 2006 | RCT | KOR | coronary angiography | 67.4 | 56.0% | 1.34 | ± | -- | 100.0% | 199.70 | 0 | 2 | 2 | 1 |
| Josephson et al., 2005 | Retrospective | USA | stroke protocol CT (a noncontrast CT scan of the brain, followed by CT perfusion imaging, followed by CTA) | - | - | - | | | - | - | 2 | 2 | 2 | 1 |
| Kandula et al., 2010 | Retrospective | USA | percutaneous coronary intervention | 72.7 | 46.5% | 1.01 | ± | -- | - | 182.10 | 2 | 2 | 2 | 1 |
| Kane et al., 2008 | Retrospective | USA | percutaneous transluminal renal angioplasty (PTRA) | 72.4 | 57.7% | 2.32 | ± | -- | 100.0% | 90.58 | 0 | 2 | 2 | 2 |
| Kapoor et al., 2002 | RCT | IND | coronary angiography | 54.5 | 88.6% | 1.19 | ± | 0.23 | - | 77.80 | 0 | 2 | 2 | 0 |
| Kapoor et al., 1996 | RCT | IND | coronary angiography | 61.4 | 85.0% | 1.52 | ± | 0.68 | - | 125.00 | 0 | 2 | 2 | 0 |
| Kashif et al., 2013 | Retrospective | PAK | coronary angiography with or without intervention | 64.0 | 72.0% | - | | | - | - | 0 | 2 | 2 | 0 |
| Kato et al., 2011 | Retrospective | JPN | carotid artery stenting | 73.7 | - | 1.11 | ± | -- | 60.0% | 147.30 | 2 | 2 | 2 | 2 |
| Kawashima et al., 2006 | Retrospective | JPN | coronary angiography or percutaneous coronary intervention | 69.4 | 83.9% | 1.68 | ± | -- | - | - | 0 | 2 | 2 | 0 |
| Khanal et al., 2005 | Retrospective | USA | percutaneous coronary intervention | 63.6 | 64.9% | 1.10 | ± | 0.61 | - | 221.00 | 2 | 2 | 2 | 1 |
| Kini et al., 2002 | Retrospective | USA | percutaneous coronary intervention | 72.0 | 70.0% | 2.21 | ± | -- | - | 159.00 | 0 | 2 | 2 | 0 |
| Komenda et al., 2007 | Case-series | CAN | cardiac or peripheral vessel angiography | 64.0 | 48.0% | 2.42 | ± | 1.39 | 100.0% | - | 0 | 2 | 2 | 2 |
| Kooiman et al., 2014 | Retrospective | USA | percutaneous coronary intervention | - | - | - | | | - | - | 2 | 2 | 2 | 1 |
| Krol et al., 2007 | Retrospective | CAN | Computed Tomography Angiography | 68.2 | 62.0% | - | | | - | - | 2 | 2 | 2 | 1 |
| Kumar et al., 2009 | Retrospective | GBR | coronary angiography | 56.3 | 64.5% | - | | | 100.0% | 55.74 | 0 | 2 | 2 | 2 |
| Kumar et al., 2017 | Prospective | IND | coronary angiography ± percutaneous coronary intervention | 56.6 | 69.6% | - | | | - | 159.50 | 2 | 2 | 2 | 0 |
| Lee et al., 2007 | RCT | TWN | coronary angiography | 65.9 | 65.0% | 4.90 | ± | 1.60 | - | 108.10 | 0 | 2 | 2 | 1 |
| Leoncini et al., 2014 | RCT | ITA | early invasive strategy (PCI) | 66.1 | 65.5% | 0.96 | ± | 0.28 | 14.7% | 172.00 | 1 | 2 | 2 | 2 |
| Leone et al., 2012 | Retrospective | ITA | percutaneous coronary intervention | 63.0 | 77.0% | 1.12 | ± | --- | 23.0% | 214.00 | 2 | 2 | 2 | 0 |
| Li et al., 2016 | Prospective | CHN | percutaneous coronary intervention | 65.0 | 81.4% | 1.14 | ± | 0.41 | 30.7% | 131.90 | 2 | 2 | 2 | 1 |
| Lin et al., 2014 | Retrospective | TWN | percutaneous coronary intervention | 62.9 | 85.5% | 1.10 | ± | 0.40 | 39.9% | 277.10 | 2 | 2 | 2 | 1 |
| Liss et al., 2006 | Retrospective | SWE | coronary angiography and percutaneous coronary intervention | 64.5 | 67.6% | - | | | - | - | 2 | 2 | 2 | 2 |
| Liu et al., 2015 | Prospective | CHN | percutaneous coronary intervention | 72.5 | 69.1% | 0.97 | ± | 0.22 | - | 150.30 | 0 | 2 | 2 | 2 |
| Liu et al., 2015 | Prospective | CHN | percutaneous coronary intervention | 70.0 | 70.7% | 1.60 | ± | -- | 100.0% | 154.21 | 0 | 2 | 2 | 1 |
| Majumdar et al., 2009 | RCT | CAN | coronary angiography | 63.0 | 65.0% | 2.90 | ± | 1.90 | - | 129.00 | 0 | 2 | 2 | 2 |
| Mandal et al., 2018 | Prospective | USA | percutaneous coronary intervention | 58.5 | 73.0% | 1.06 | ± | -- | - | 160.30 | 2 | 2 | 2 | 0 |
| Manske et al., 1990 | Prospective | USA | coronary angiography | 37.0 |  | 5.90 | ± | 1.59 | - | - | 0 | 1 | 2 | 1 |
| Marenzi et al., 2009 | Prospective | ITA | percutaneous coronary intervention | 62.2 | 81.3% | 1.09 | ± | 0.27 | - | 264.43 | 2 | 2 | 2 | 1 |
| Marenzi et al., 2006 | RCT | ITA | primary angioplasty | 62.6 | 82.0% | 1.06 | ± | -- | - | 274.00 | 2 | 2 | 2 | 1 |
| Marenzi et al., 2003 | RCT | ITA | coronary intervention | 69.0 | 77.0% | 3.10 | ± | 1.00 | - | 258.00 | 0 | 2 | 2 | 2 |
| Martin-Paredero et al., 1983 | Retrospective | USA | major angiography | - | 58.8% | - | | | - | - | 2 | 2 | 2 | 1 |
| Masuda et al., 2008 | RCT | JPN | coronary procedures | 76.0 | 59.0% | 1.32 | ± | 0.65 | - | 120.00 | 0 | 2 | 2 | 2 |
| Matejka et al., 2010 | RCT | CZE | cardiac angiography and/or angioplasty | 75.0 | 64.0% | 2.06 | ± | 0.59 | 100.0% | 94.00 | 0 | 2 | 2 | 0 |
| McCullough et al., 2019 | Retrospective | USA | interventional cardiovascular procedure | 64.9 | 64.0% | - | | | - | - | 2 | 1 | 2 | 1 |
| Meloni et al., 2017 | Prospective | ITA | transluminal angioplasty of lower limb | 71.1 | 61.2% | - | | | - | - | 0 | 2 | 2 | 2 |
| Miner et al., 2004 | RCT | CAN | percutaneous coronary intervention | 69.0 | 66.0% | 1.47 | ± | 0.66 | - | 350.00 | 0 | 2 | 2 | 2 |
| Morabito et al., 2012 | Prospective | ITA | coronary angiography or percutaneous coronary intervention | 65.8 | 74.4% | 1.12 | ± | 0.33 | 34.2% | 220.10 | 2 | 2 | 2 | 2 |
| Mueller et al., 2005 | Prospective | CHE | percutaneous coronary intervention | 64.0 | 75.0% | 0.91 | ± | -- | 10.0% | 226.00 | 2 | 2 | 2 | 0 |
| Mueller-Lenke et al., 2008 | Prospective | CHE | percutaneous coronary intervention | 64.0 | 74.0% | 0.93 | ± | 0.28 | 11.2% | 237.21 | 2 | 2 | 2 | 0 |
| Negighi et al., 2019 | Prospective | JPN | percutaneous coronary intervention | 76.6 | 65.0% | 2.46 | ± | 1.16 | 100.0% | 72.00 | 0 | 2 | 2 | 2 |
| Neyra et al., 2013 | Retrospective | USA | coronary angiography | 59.7 | 60.3% | 0.92 | ± | -- | 0.0% | 142.67 | 2 | 2 | 2 | 2 |
| Nikolsky et al., 2004 | Prospective | USA | percutaneous coronary intervention | 62.4 | 65.9% | 0.93 | ± | 0.17 |  | 267.00 | 0 | 2 | 2 | 2 |
| Oldemeyer et al., 2003 | RCT | USA | angiography | 75.0 | 55.0% | 1.66 | ± | 0.65 | 100.0% | 127.00 | 0 | 2 | 2 | 0 |
| Ozturk et al., 2016 | Prospective | TUR | percutaneous coronary intervention | 61.5 | - | - | | | - | - | 2 | 2 | 2 | 2 |
| Pakfetrat et al., 2010 | Prospective | IRN | coronary angiography or percutaneous coronary intervention | 58.3 | - | 1.10 | ± | 0.20 | 30.3% | 64.80 | 2 | 2 | 2 | 0 |
| Reed et al., 2010 | Prospective | USA | percutaneous coronary intervention | 66.0 | 63.6% | 1.23 | ± | -- | - | 207.00 | 2 | 2 | 2 | 1 |
| Reinecke et al., 2011 | RCT | DEU | coronary angiography | 66.7 | 82.9% | 1.40 | ± | -- | 100.0% | 188.00 | 0 | 2 | 2 | 2 |
| Rey et al., 2011 | Retrospective | ESP | percutaneous coronary intervention | 73.0 | 75.0% | 2.00 | ± | 0.60 | - | 368.00 | 0 | 2 | 2 | 2 |
| Rigatelli et al., 2004 | Retrospective | ITA | coronary and aortoiliac angiography | 68.4 | 72.3% | - | | | - | - | 2 | 2 | 2 | 1 |
| Rozenbaum et al., 2018 | Prospective | ISR | coronary procedures | 71.4 | 53.3% | 1.85 | ± | -- | 100.0% | - | 0 | 2 | 1 | 2 |
| Sedhai et al., 2017 | Retrospective | USA | cardiac catheterization | 69.1 | 55.0% | - | | | - | 109.50 | 2 | 2 | 2 | 1 |
| Shams Eddine Taher et al., 2015 | Prospective | EGY | coronary angiography or percutaneous coronary intervention | 55.5 | 78.0% | 0.90 | ± | 0.30 | - | 157.00 | 2 | 2 | 2 | 1 |
| Silvain et al., 2018 | Prospective | FRA | percutaneous coronary intervention | 62.9 | 75.8% | 0.90 | ± | 0.55 | - | 213.90 | 1 | 2 | 2 | 2 |
| Soofi et al., 2006 | Retrospective | PAK | cardiac catheterization and percutaneous coronary intervention | 57.0 | 71.4% | - | | | - | - | 2 | 2 | 2 | 1 |
| Srodon et al., 2003 | Retrospective | GBR | lower limb angiogram and angioplasty procedures | 70.0 | - | 1.20 | ± | -- | - | - | 2 | 2 | 1 | 1 |
| Stevens et al., 1999 | RCT | USA | percutaneous coronary intervention | 69.6 | 56.0% | 2.55 | ± | 0.91 | - | 161.50 | 0 | 2 | 2 | 1 |
| Stone et al., 2003 | RCT | USA | diagnostic and/or interventional cardiology procedures | 70.2 | 66.3% | 1.81 | ± | 0.83 | 100.0% | 162.00 | 0 | 2 | 2 | 2 |
| Tadros et al., 2005 | Retrospective | USA | cardiac catheterization | 60.1 | 63.2% | 2.90 | ± | 1.50 | 100.0% | 84.30 | 0 | 2 | 2 | 1 |
| Tanaka et al., 2011 | RCT | JPN | primary angioplasty | 60.5 | 82.0% | 0.80 | ± | 0.19 | - | 216.00 | 1 | 2 | 2 | 1 |
| Tasanarong et al., 2013 | RCT | THA | coronary procedures | 66.0 | 70.3% | 1.63 | ± | 0.53 | 100.0% | 134.00 | 0 | 2 | 2 | 1 |
| Toso et al., 2010 | RCT | ITA | coronary angiography and/or angioplasty | 76.0 | 60.0% | 1.18 | ± | 0.33 | 100.0% | 164.00 | 0 | 2 | 2 | 2 |
| Uyarel et al., 2009 | Retrospective | TUR | primary angioplasty | 56.5 | 82.9% | 0.97 | ± | 0.30 | 11.8% | 237.02 | 2 | 2 | 2 | 2 |
| Valappil et al., 2018 | Prospective | IND | percutaneous coronary intervention | 61.8 | 83.0% | - | | | 100.0% | 206.40 | 0 | 2 | 2 | 1 |
| Valappil et al., 2018 | RCT | IND | coronary angioplasty | 60.5 | 80.0% | 1.40 | ± | -- | 100.0% | - | 0 | 2 | 2 | 1 |
| Vasheghani-Farahani et al., 2009 | RCT | IRN | coronary angiography | 63.8 | 81.5% | 1.66 | ± | 0.50 | 100.0% | 113.20 | 0 | 2 | 2 | 0 |
| Webb et al., 2004 | RCT | CAN | cardiac catheterization | 70.0 | 62.0% | 1.42 | ± | -- | - | 120.00 | 0 | 2 | 2 | 0 |
| Weisbord et al., 2018 | RCT | USA | angiography | 69.6 | 93.0% | 1.50 | ± | -- | 100.0% | 85.00 | 0 | 2 | 2 | 2 |
| Weisbord et al., 2017 | Prospective | USA | coronary angiography | 67.0 | 93.0% | 1.70 | ± | 1.30 | 100.0% | - | 0 | 2 | 2 | 2 |
| Xu et al., 2019 | Prospective | CHN | percutaneous coronary intervention | 56.7 | 78.8% | 1.04 | ± | -- | 12.6% | 121.64 | 2 | 2 | 2 | 1 |
| Yang et al., 2018 | RCT | CHN | percutaneous coronary intervention | 67.8 | 72.0% | 1.10 | ± | 0.13 | - | 148.50 | 0 | 2 | 2 | 1 |
| Yoon et al., 2011 | Prospective | KOR | percutaneous coronary intervention | 66.3 | 56.3% | 1.50 | ± | -- | - | 210.93 | 2 | 2 | 2 | 0 |
| Zhou et al., 2018 | RCT | CHN | percutaneous coronary intervention | 69.1 | 61.4% | 1.06 | ± | 0.22 | - | 108.82 | 1 | 1 | 2 | 2 |

$: 0: low quality; 1: uncertain; 2: high quality.

# Included studies list

1.Investigators ACT. Acetylcysteine for prevention of renal outcomes in patients undergoing coronary and peripheral vascular angiography: main results from the randomized Acetylcysteine for Contrast-induced nephropathy Trial (ACT). Circulation. (2011) 124: 1250-9.

2.Abaci O, Arat Ozkan A, Kocas C, Cetinkal G, Sukru Karaca O, Baydar O, et al. Impact of Rosuvastatin on contrast-induced acute kidney injury in patients at high risk for nephropathy undergoing elective angiography. Am J Cardiol. (2015) 115: 867-71.

3.Abizaid AS, Clark CE, Mintz GS, Dosa S, Popma JJ, Pichard AD, et al. Effects of dopamine and aminophylline on contrast-induced acute renal failure after coronary angioplasty in patients with preexisting renal insufficiency. Am J Cardiol. (1999) 83: 260-3, a5.

4.Agrawal V, Swami A, Kosuri R, Alsabbagh M, Agarwal M, Samarapungavan D, et al. Contrast-induced acute kidney injury in renal transplant recipients after cardiac catheterization. Clin Nephrol. (2009) 71: 687-96.

5.Aguiar-Souto P, Ferrante G, Del Furia F, Barlis P, Khurana R, and Di Mario C. Frequency and predictors of contrast-induced nephropathy after angioplasty for chronic total occlusions. Int J Cardiol. (2010) 139: 68-74.

6.Al Adas Z, Lodewyk K, Robinson D, Qureshi S, Kabbani LS, Sullivan B, et al. Contrast-induced nephropathy after peripheral vascular intervention: Long-term renal outcome and risk factors for progressive renal dysfunction. J Vasc Surg. (2019) 69: 913-20.

7.Allaqaband S, Tumuluri R, Malik AM, Gupta A, Volkert P, Shalev Y, et al. Prospective randomized study of N-acetylcysteine, fenoldopam, and saline for prevention of radiocontrast-induced nephropathy. Catheter Cardiovasc Interv. (2002) 57: 279-83.

8.Alli OO, Singh IM, Holmes DR, Jr., Pulido JN, Park SJ, and Rihal CS. Percutaneous left ventricular assist device with TandemHeart for high-risk percutaneous coronary intervention: the Mayo Clinic experience. Catheter Cardiovasc Interv. (2012) 80: 728-34.

9.Asif A, Cherla G, Merrill D, Cipleu CD, Tawakol JB, Epstein DL, et al. Venous mapping using venography and the risk of radiocontrast-induced nephropathy. Semin Dial. (2005) 18: 239-42.

10.Attallah N, Yassine L, Musial J, Yee J, and Fisher K. The potential role of statins in contrast nephropathy. Clin Nephrol. (2004) 62: 273-8.

11.Azzalini L, Vilca LM, Lombardo F, Poletti E, Laricchia A, Beneduce A, et al. Incidence of contrast-induced acute kidney injury in a large cohort of all-comers undergoing percutaneous coronary intervention: Comparison of five contrast media. Int J Cardiol. (2018) 273: 69-73.

12.Bolognese L, Falsini G, Schwenke C, Grotti S, Limbruno U, Liistro F, et al. Impact of iso-osmolar versus low-osmolar contrast agents on contrast-induced nephropathy and tissue reperfusion in unselected patients with ST-segment elevation myocardial infarction undergoing primary percutaneous coronary intervention (from the Contrast Media and Nephrotoxicity Following Primary Angioplasty for Acute Myocardial Infarction [CONTRAST-AMI] Trial). Am J Cardiol. (2012) 109: 67-74.

13.Boscheri A, Weinbrenner C, Botzek B, Reynen K, Kuhlisch E, and Strasser RH. Failure of ascorbic acid to prevent contrast-media induced nephropathy in patients with renal dysfunction. Clin Nephrol. (2007) 68: 279-86.

14.Brar SS, Shen AY, Jorgensen MB, Kotlewski A, Aharonian VJ, Desai N, et al. Sodium bicarbonate vs sodium chloride for the prevention of contrast medium-induced nephropathy in patients undergoing coronary angiography: a randomized trial. Jama. (2008) 300: 1038-46.

15.Briguori C, Manganelli F, Scarpato P, Elia PP, Golia B, Riviezzo G, et al. Acetylcysteine and contrast agent-associated nephrotoxicity. J Am Coll Cardiol. (2002) 40: 298-303.

16.Briguori C, Visconti G, Rivera NV, Focaccio A, Golia B, Giannone R, et al. Cystatin C and contrast-induced acute kidney injury. Circulation. (2010) 121: 2117-22.

17.Brown JR, DeVries JT, Piper WD, Robb JF, Hearne MJ, Ver Lee PM, et al. Serious renal dysfunction after percutaneous coronary interventions can be predicted. Am Heart J. (2008) 155: 260-6.

18.Budano C, Levis M, D'Amico M, Usmiani T, Fava A, Sbarra P, et al. Impact of contrast-induced acute kidney injury definition on clinical outcomes. Am Heart J. (2011) 161: 963-71.

19.Budhiraja P, Chen Z, and Popovtzer M. Sodium bicarbonate versus normal saline for protection against contrast nephropathy. Ren Fail. (2009) 31: 118-23.

20.Carbonell N, Blasco M, Sanjuán R, Pérez-Sancho E, Sanchis J, Insa L, et al. Intravenous N-acetylcysteine for preventing contrast-induced nephropathy: a randomised trial. Int J Cardiol. (2007) 115: 57-62.

21.Carbonell N, Sanjuán R, Blasco M, Jordá A, and Miguel A. N-acetylcysteine: short-term clinical benefits after coronary angiography in high-risk renal patients. Rev Esp Cardiol. (2010) 63: 12-9.

22.Caruso M, Balasus F, Incalcaterra E, Ruggieri A, Evola S, Fattouch K, et al. Contrast-induced nephropathy after percutaneous coronary intervention in simple lesions: risk factors and incidence are affected by the definition utilized. Intern Med. (2011) 50: 983-9.

23.Cochran ST, Wong WS, and Roe DJ. Predicting angiography-induced acute renal function impairment: clinical risk model. AJR Am J Roentgenol. (1983) 141: 1027-33.

24.D'Elia JA, Gleason RE, Alday M, Malarick C, Godley K, Warram J, et al. Nephrotoxicity from angiographic contrast material. A prospective study. Am J Med. (1982) 72: 719-25.

25.Dittrich R, Akdeniz S, Kloska SP, Fischer T, Ritter MA, Seidensticker P, et al. Low rate of contrast-induced Nephropathy after CT perfusion and CT angiography in acute stroke patients. J Neurol. (2007) 254: 1491-7.

26.Dvoršak B, Kanič V, Ekart R, Bevc S, and Hojs R. Ascorbic Acid for the prevention of contrast-induced nephropathy after coronary angiography in patients with chronic renal impairment: a randomized controlled trial. Ther Apher Dial. (2013) 17: 384-90.

27.Eisenhart E, Benson S, Lacombe P, Himmelfarb J, Zimmerman R, Schimelman B, et al. Safety of low volume iodinated contrast administration for arteriovenous fistula intervention in chronic kidney disease stage 4 or 5 utilizing a bicarbonate prophylaxis strategy. Semin Dial. (2010) 23: 638-42.

28.Er F, Nia AM, Dopp H, Hellmich M, Dahlem KM, Caglayan E, et al. Ischemic preconditioning for prevention of contrast medium-induced nephropathy: randomized pilot RenPro Trial (Renal Protection Trial). Circulation. (2012) 126: 296-303.

29.Farhan S, Vogel B, Tentzeris I, Jarai R, Freynhofer MK, Smetana P, et al. Contrast induced acute kidney injury in acute coronary syndrome patients: A single centre experience. Eur Heart J Acute Cardiovasc Care. (2016) 5: 55-61.

30.Ferrario F, Barone MT, Landoni G, Genderini A, Heidemperger M, Trezzi M, et al. Acetylcysteine and non-ionic isosmolar contrast-induced nephropathy--a randomized controlled study. Nephrol Dial Transplant. (2009) 24: 3103-7.

31.Firouzi A, Eshraghi A, Shakerian F, Sanati HR, Salehi N, Zahedmehr A, et al. Efficacy of pentoxifylline in prevention of contrast-induced nephropathy in angioplasty patients. Int Urol Nephrol. (2012) 44: 1145-9.

32.Firouzi A, Maadani M, Kiani R, Shakerian F, Sanati HR, Zahedmehr A, et al. Intravenous magnesium sulfate: new method in prevention of contrast-induced nephropathy in primary percutaneous coronary intervention. Int Urol Nephrol. (2015) 47: 521-5.

33.Frank H, Werner D, Lorusso V, Klinghammer L, Daniel WG, Kunzendorf U, et al. Simultaneous hemodialysis during coronary angiography fails to prevent radiocontrast-induced nephropathy in chronic renal failure. Clin Nephrol. (2003) 60: 176-82.

34.Ge ML, Han YL, Huang L, Yang LX, Yu B, Zhao RP, et al. Effect and safety of rosuvastatin for prevention of contrast-induced acute kidney injury after percutaneous coronary intervention in patients with diabetes associated with mild-moderate renal insufficiency. Medical journal of chinese people's liberation army. (2014) 39: 277‐82.

35.Ghani AA, Hussain N, and Al Helal B. Can continuous venovenous hemofiltration prevent contrast-agent induced nephropathy in patients with advanced chronic kidney disease after coronary angiography? Saudi J Kidney Dis Transpl. (2011) 22: 54-60.

36.Gomes AS, Baker JD, Martin-Paredero V, Dixon SM, Takiff H, Machleder HI, et al. Acute renal dysfunction after major arteriography. AJR Am J Roentgenol. (1985) 145: 1249-53.

37.Gomes VO, Poli de Figueredo CE, Caramori P, Lasevitch R, Bodanese LC, Araújo A, et al. N-acetylcysteine does not prevent contrast induced nephropathy after cardiac catheterisation with an ionic low osmolality contrast medium: a multicentre clinical trial. Heart. (2005) 91: 774-8.

38.Grossman PM, Ali SS, Aronow HD, Boros M, Nypaver TJ, Schreiber TL, et al. Contrast-induced nephropathy in patients undergoing endovascular peripheral vascular intervention: Incidence, risk factors, and outcomes as observed in the Blue Cross Blue Shield of Michigan Cardiovascular Consortium. J Interv Cardiol. (2017) 30: 274-80.

39.Grossman PM, Gurm HS, McNamara R, Lalonde T, Changezi H, Share D, et al. Percutaneous coronary intervention complications and guide catheter size: bigger is not better. JACC Cardiovasc Interv. (2009) 2: 636-44.

40.Gurm HS, Dixon SR, Smith DE, Share D, Lalonde T, Greenbaum A, et al. Renal function-based contrast dosing to define safe limits of radiographic contrast media in patients undergoing percutaneous coronary interventions. J Am Coll Cardiol. (2011) 58: 907-14.

41.Gurm HS, Smith DE, Berwanger O, Share D, Schreiber T, Moscucci M, et al. Contemporary use and effectiveness of N-acetylcysteine in preventing contrast-induced nephropathy among patients undergoing percutaneous coronary intervention. JACC Cardiovasc Interv. (2012) 5: 98-104.

42.Haque AS, Rabbani MG, Hossain MR, Rashid MH, Mostafi M, and Rahman AMJJoM. Contrast-Induced Nephropathy in 118 Coronary Angiographic Cases. (2009) 10: 94-96.

43.Hassan AKM, Shams-Eddin H, Abdel-Rahim MHM, El-Hafeez HAA, and Edroos S. Cardio- and reno-protective effect of remote ischemic preconditioning in patients undergoing percutaneous coronary intervention. A prospective, non-randomized controlled trial. The Egyptian Heart Journal. (2015) 67: 315-23.

44.Hekimian G, Kim M, Passefort S, Duval X, Wolff M, Leport C, et al. Preoperative use and safety of coronary angiography for acute aortic valve infective endocarditis. Heart. (2010) 96: 696-700.

45.Hobikoğlu GF, Norgaz T, Aksu H, Özer O, Sarı İ, Karabulut A, et al. The effectiveness of oral N-acetylcysteine administered three hours before angiography in the prevention of contrast nephropathy in patients with mildly elevated creatinine levels. (2007) 35: 9-12.

46.Hopyan JJ, Gladstone DJ, Mallia G, Schiff J, Fox AJ, Symons SP, et al. Renal safety of CT angiography and perfusion imaging in the emergency evaluation of acute stroke. AJNR Am J Neuroradiol. (2008) 29: 1826-30.

47.Hoshi T, Sato A, Kakefuda Y, Harunari T, Watabe H, Ojima E, et al. Preventive effect of statin pretreatment on contrast-induced acute kidney injury in patients undergoing coronary angioplasty: propensity score analysis from a multicenter registry. Int J Cardiol. (2014) 171: 243-9.

48.Hsieh YC, Ting CT, Liu TJ, Wang CL, Chen YT, and Lee WL. Short- and long-term renal outcomes of immediate prophylactic hemodialysis after cardiovascular catheterizations in patients with severe renal insufficiency. Int J Cardiol. (2005) 101: 407-13.

49.Jabara R, Gadesam RR, Pendyala LK, Knopf WD, Chronos N, Chen JP, et al. Impact of the definition utilized on the rate of contrast-induced nephropathy in percutaneous coronary intervention. Am J Cardiol. (2009) 103: 1657-62.

50.James MT, Ghali WA, Knudtson ML, Ravani P, Tonelli M, Faris P, et al. Associations between acute kidney injury and cardiovascular and renal outcomes after coronary angiography. Circulation. (2011) 123: 409-16.

51.Jarocka IT, Bachórzewska-Gajewska H, Kobus G, Czaban S, Małyszko J, and Dobrzycki S. Renal function after percutaneous coronary interventions depending on the type of hydration. Adv Med Sci. (2013) 58: 369-75.

52.Jo SH, Youn TJ, Koo BK, Park JS, Kang HJ, Cho YS, et al. Renal toxicity evaluation and comparison between visipaque (iodixanol) and hexabrix (ioxaglate) in patients with renal insufficiency undergoing coronary angiography: the RECOVER study: a randomized controlled trial. J Am Coll Cardiol. (2006) 48: 924-30.

53.Josephson SA, Dillon WP, and Smith WS. Incidence of contrast nephropathy from cerebral CT angiography and CT perfusion imaging. Neurology. (2005) 64: 1805-6.

54.Kandula P, Shah R, Singh N, Markwell SJ, Bhensdadia N, and Navaneethan SD. Statins for prevention of contrast-induced nephropathy in patients undergoing non-emergent percutaneous coronary intervention. Nephrology (Carlton). (2010) 15: 165-70.

55.Kane GC, Stanson AW, Kalnicka D, Rosenthal DW, Lee CU, Textor SC, et al. Comparison between gadolinium and iodine contrast for percutaneous intervention in atherosclerotic renal artery stenosis: clinical outcomes. Nephrol Dial Transplant. (2008) 23: 1233-40.

56.Kapoor A, Kumar S, Gulati S, Gambhir S, Sethi RS, and Sinha N. The role of theophylline in contrast-induced nephropathy: a case-control study. Nephrol Dial Transplant. (2002) 17: 1936-41.

57.Kapoor A, Sinha N, Sharma RK, Shrivastava S, Radhakrishnan S, Goel PK, et al. Use of dopamine in prevention of contrast induced acute renal failure--a randomised study. Int J Cardiol. (1996) 53: 233-6.

58.Kashif W, Khawaja A, Yaqub S, and Hussain SA. Clinically significant contrast induced acute kidney injury after non-emergent cardiac catheterization--risk factors and impact on length of hospital stay. J Coll Physicians Surg Pak. (2013) 23: 842-7.

59.Kato T, Sakai H, Tsujimoto M, and Nishimura Y. Prolonged carotid sinus reflex is a risk factor for contrast-induced nephropathy following carotid artery stenting. AJNR Am J Neuroradiol. (2011) 32: 441-5.

60.Kawashima S, Takano H, Iino Y, Takayama M, and Takano T. Prophylactic hemodialysis does not prevent contrast-induced nephropathy after cardiac catheterization in patients with chronic renal insufficiency. Circ J. (2006) 70: 553-8.

61.Khanal S, Attallah N, Smith DE, Kline-Rogers E, Share D, O'Donnell MJ, et al. Statin therapy reduces contrast-induced nephropathy: an analysis of contemporary percutaneous interventions. Am J Med. (2005) 118: 843-9.

62.Kini AS, Mitre CA, Kim M, Kamran M, Reich D, and Sharma SK. A protocol for prevention of radiographic contrast nephropathy during percutaneous coronary intervention: effect of selective dopamine receptor agonist fenoldopam. Catheter Cardiovasc Interv. (2002) 55: 169-73.

63.Komenda P, Zalunardo N, Burnett S, Love J, Buller C, Taylor P, et al. Conservative outpatient renoprotective protocol in patients with low GFR undergoing contrast angiography: a case series. Clin Exp Nephrol. (2007) 11: 209-13.

64.Kooiman J, Seth M, Share D, Dixon S, and Gurm HS. The association between contrast dose and renal complications post PCI across the continuum of procedural estimated risk. PLoS One. (2014) 9: e90233.

65.Krol AL, Dzialowski I, Roy J, Puetz V, Subramaniam S, Coutts SB, et al. Incidence of radiocontrast nephropathy in patients undergoing acute stroke computed tomography angiography. Stroke. (2007) 38: 2364-6.

66.Kumar N, Dahri L, Brown W, Duncan N, Singh S, Baker C, et al. Effect of elective coronary angiography on glomerular filtration rate in patients with advanced chronic kidney disease. Clin J Am Soc Nephrol. (2009) 4: 1907-13.

67.Kumar S, Nair RK, Aggarwal N, Abbot AK, Muthukrishnan J, and Kumar KV. Risk factors for contrast-induced nephropathy after coronary angiography. Saudi J Kidney Dis Transpl. (2017) 28: 318-24.

68.Lee PT, Chou KJ, Liu CP, Mar GY, Chen CL, Hsu CY, et al. Renal protection for coronary angiography in advanced renal failure patients by prophylactic hemodialysis. A randomized controlled trial. J Am Coll Cardiol. (2007) 50: 1015-20.

69.Leoncini M, Toso A, Maioli M, Tropeano F, Villani S, and Bellandi F. Early high-dose rosuvastatin for contrast-induced nephropathy prevention in acute coronary syndrome: Results from the PRATO-ACS Study (Protective Effect of Rosuvastatin and Antiplatelet Therapy On contrast-induced acute kidney injury and myocardial damage in patients with Acute Coronary Syndrome). J Am Coll Cardiol. (2014) 63: 71-9.

70.Leone AM, De Caterina AR, Sciahbasi A, Aurelio A, Basile E, Porto I, et al. Sodium bicarbonate plus N-acetylcysteine to prevent contrast-induced nephropathy in primary and rescue percutaneous coronary interventions: the BINARIO (BIcarbonato e N-Acetil-cisteina nell'infaRto mIocardico acutO) study. EuroIntervention. (2012) 8: 839-47.

71.Li H, Huang S, He Y, Liu Y, Liu Y, Chen J, et al. Impact of an Early Decrease in Systolic Blood Pressure on The Risk of Contrast-Induced Nephropathy after Percutaneous Coronary Intervention. Heart Lung Circ. (2016) 25: 118-23.

72.Lin YS, Fang HY, Hussein H, Fang CY, Chen YL, Hsueh SK, et al. Predictors of contrast-induced nephropathy in chronic total occlusion percutaneous coronary intervention. EuroIntervention. (2014) 9: 1173-80.

73.Liss P, Persson PB, Hansell P, and Lagerqvist B. Renal failure in 57 925 patients undergoing coronary procedures using iso-osmolar or low-osmolar contrast media. Kidney Int. (2006) 70: 1811-7.

74.Liu Y, Liu YH, Chen JY, Tan N, Zhou YL, Duan CY, et al. Safe contrast volumes for preventing contrast-induced nephropathy in elderly patients with relatively normal renal function during percutaneous coronary intervention. Medicine (Baltimore). (2015) 94: e615.

75.Liu YH, Liu Y, Tan N, Chen JY, Zhou YL, Luo JF, et al. Contrast-induced nephropathy following chronic total occlusion percutaneous coronary intervention in patients with chronic kidney disease. Eur Radiol. (2015) 25: 2274-81.

76.Majumdar SR, Kjellstrand CM, Tymchak WJ, Hervas-Malo M, Taylor DA, and Teo KK. Forced euvolemic diuresis with mannitol and furosemide for prevention of contrast-induced nephropathy in patients with CKD undergoing coronary angiography: a randomized controlled trial. Am J Kidney Dis. (2009) 54: 602-9.

77.Mandal A, Paudel MS, Kafle P, Khalid M, Bhattarai B, Kanth R, et al. Contrast-induced Nephropathy Following Percutaneous Coronary Intervention at a Tertiary Cardiac Center in Nepal. Cureus. (2018) 10: e3331.

78.Manske CL, Sprafka JM, Strony JT, and Wang Y. Contrast nephropathy in azotemic diabetic patients undergoing coronary angiography. Am J Med. (1990) 89: 615-20.

79.Marenzi G, Assanelli E, Campodonico J, Lauri G, Marana I, De Metrio M, et al. Contrast volume during primary percutaneous coronary intervention and subsequent contrast-induced nephropathy and mortality. Ann Intern Med. (2009) 150: 170-7.

80.Marenzi G, Assanelli E, Marana I, Lauri G, Campodonico J, Grazi M, et al. N-acetylcysteine and contrast-induced nephropathy in primary angioplasty. N Engl J Med. (2006) 354: 2773-82.

81.Marenzi G, Marana I, Lauri G, Assanelli E, Grazi M, Campodonico J, et al. The prevention of radiocontrast-agent-induced nephropathy by hemofiltration. N Engl J Med. (2003) 349: 1333-40.

82.Martin-Paredero V, Dixon SM, Baker JD, Takiff H, Gomes AS, Busuttil RW, et al. Risk of renal failure after major angiography. Arch Surg. (1983) 118: 1417-20.

83.Masuda M, Yamada T, Okuyama Y, Morita T, Sanada S, Furukawa Y, et al. Sodium bicarbonate improves long-term clinical outcomes compared with sodium chloride in patients with chronic kidney disease undergoing an emergent coronary procedure. Circ J. (2008) 72: 1610-4.

84.Matejka J, Varvarovsky I, Vojtisek P, Herman A, Rozsival V, Borkova V, et al. Prevention of contrast-induced acute kidney injury by theophylline in elderly patients with chronic kidney disease. Heart Vessels. (2010) 25: 536-42.

85.McCullough PA, Todoran TM, Brilakis ES, Ryan MP, and Gunnarsson C. Rate of major adverse renal or cardiac events with iohexol compared to other low osmolar contrast media during interventional cardiovascular procedures. Catheter Cardiovasc Interv. (2019) 93: E90-e97.

86.Meloni M, Giurato L, Izzo V, Stefanini M, Gandini R, and Uccioli L. Risk of contrast induced nephropathy in diabetic patients affected by critical limb ischemia and diabetic foot ulcers treated by percutaneous transluminal angioplasty of lower limbs. Diabetes Metab Res Rev. (2017) 33.

87.Miner SE, Dzavik V, Nguyen-Ho P, Richardson R, Mitchell J, Atchison D, et al. N-acetylcysteine reduces contrast-associated nephropathy but not clinical events during long-term follow-up. Am Heart J. (2004) 148: 690-5.

88.Morabito S, Pistolesi V, Benedetti G, Di Roma A, Colantonio R, Mancone M, et al. Incidence of contrast-induced acute kidney injury associated with diagnostic or interventional coronary angiography. J Nephrol. (2012) 25: 1098-107.

89.Mueller C, Seidensticker P, Buettner HJ, Perruchoud AP, Staub D, Christ A, et al. Incidence of contrast nephropathy in patients receiving comprehensive intravenous and oral hydration. Swiss Med Wkly. (2005) 135: 286-90.

90.Mueller-Lenke N, Buerkle G, Klima T, Breidthardt T, Buettner HJ, and Mueller C. Incidence of contrast-induced nephropathy with volume supplementation--insights from a large cohort. Med Princ Pract. (2008) 17: 409-14.

91.Negishi Y, Tanaka A, Ishii H, Takagi K, Inoue Y, Uemura Y, et al. Contrast-Induced Nephropathy and Long-Term Clinical Outcomes Following Percutaneous Coronary Intervention in Patients With Advanced Renal Dysfunction (Estimated Glomerular Filtration Rate <30 ml/min/1.73 m(2)). Am J Cardiol. (2019) 123: 361-67.

92.Neyra JA, Shah S, Mooney R, Jacobsen G, Yee J, and Novak JE. Contrast-induced acute kidney injury following coronary angiography: a cohort study of hospitalized patients with or without chronic kidney disease. Nephrol Dial Transplant. (2013) 28: 1463-71.

93.Nikolsky E, Mehran R, Turcot D, Aymong ED, Mintz GS, Lasic Z, et al. Impact of chronic kidney disease on prognosis of patients with diabetes mellitus treated with percutaneous coronary intervention. Am J Cardiol. (2004) 94: 300-5.

94.Oldemeyer JB, Biddle WP, Wurdeman RL, Mooss AN, Cichowski E, and Hilleman DE. Acetylcysteine in the prevention of contrast-induced nephropathy after coronary angiography. Am Heart J. (2003) 146: E23.

95.Ozturk D, Celik O, Erturk M, Kalkan AK, Uzun F, Akturk IF, et al. Utility of the Logistic Clinical Syntax Score in the Prediction of Contrast-Induced Nephropathy After Primary Percutaneous Coronary Intervention. Can J Cardiol. (2016) 32: 240-6.

96.Pakfetrat M, Nikoo MH, Malekmakan L, Tabande M, Roozbeh J, Ganbar Ali RJ, et al. Comparison of risk factors for contrast-induced acute kidney injury between patients with and without diabetes. Hemodial Int. (2010) 14: 387-92.

97.Reed MC, Moscucci M, Smith DE, Share D, LaLonde T, Mahmood SA, et al. The relative renal safety of iodixanol and low-osmolar contrast media in patients undergoing percutaneous coronary intervention. Insights from Blue Cross Blue Shield of Michigan Cardiovascular Consortium (BMC2). J Invasive Cardiol. (2010) 22: 467-72.

98.Reinecke H, Fobker M, Wellmann J, Becke B, Fleiter J, Heitmeyer C, et al. A randomized controlled trial comparing hydration therapy to additional hemodialysis or N-acetylcysteine for the prevention of contrast medium-induced nephropathy: the Dialysis-versus-Diuresis (DVD) Trial. Clin Res Cardiol. (2007) 96: 130-9.

99.Rey JR, Iglesias D, López De Sá E, Armada E, Moreno R, Salvador O, et al. Prevention of contrast-induced nephropathy with haemofiltration in high-risk patients after percutaneous coronary intervention. Acute Card Care. (2011) 13: 164-9.

100.Rigatelli G. Aortoiliac angiography during coronary artery angiography detects significant occult aortoiliac and renal artery atherosclerosis in patients with coronary atherosclerosis. Int J Cardiovasc Imaging. (2004) 20: 299-303.

101.Rozenbaum Z, Benchetrit S, Rozenbaum E, Neumark E, Mosseri M, and Pereg D. Ultra-Low Contrast Volume for Patients with Advanced Chronic Kidney Disease Undergoing Coronary Procedures. Nephron. (2018) 138: 296-302.

102.Sedhai YR, Golamari R, Timalsina S, Basnyat S, Koirala A, Asija A, et al. Contrast-Induced Nephropathy After Cardiac Catheterization: Culprits, Consequences and Predictors. Am J Med Sci. (2017) 354: 462-66.

103.Shams-Eddin Taher H, Hassan AKM, Dimitry SR, and Mahfouz AK. Predicting contrast induced nephropathy post coronary intervention: A prospective cohort study. The Egyptian Heart Journal. (2015) 67: 337-43.

104.Silvain J, Nguyen LS, Spagnoli V, Kerneis M, Guedeney P, Vignolles N, et al. Contrast-induced acute kidney injury and mortality in ST elevation myocardial infarction treated with primary percutaneous coronary intervention. Heart. (2018) 104: 767-72.

105.Soofi MAJPJMS. Frequency of acute renal failure after cardiac catheterization and percutaneous intervention. (2006) 22: 446-50.

106.Srodon P, Matson M, and Ham R. Contrast nephropathy in lower limb angiography. Ann R Coll Surg Engl. (2003) 85: 187-91.

107.Stevens MA, McCullough PA, Tobin KJ, Speck JP, Westveer DC, Guido-Allen DA, et al. A prospective randomized trial of prevention measures in patients at high risk for contrast nephropathy: results of the P.R.I.N.C.E. Study. Prevention of Radiocontrast Induced Nephropathy Clinical Evaluation. J Am Coll Cardiol. (1999) 33: 403-11.

108.Stone GW, McCullough PA, Tumlin JA, Lepor NE, Madyoon H, Murray P, et al. Fenoldopam mesylate for the prevention of contrast-induced nephropathy: a randomized controlled trial. Jama. (2003) 290: 2284-91.

109.Tadros GM, Malik JA, Manske CL, Kasiske BL, Dickinson SE, Herzog CA, et al. Iso-osmolar radio contrast iodixanol in patients with chronic kidney disease. J Invasive Cardiol. (2005) 17: 211-5.

110.Tanaka A, Suzuki Y, Suzuki N, Hirai T, Yasuda N, Miki K, et al. Does N-acetylcysteine reduce the incidence of contrast-induced nephropathy and clinical events in patients undergoing primary angioplasty for acute myocardial infarction? Intern Med. (2011) 50: 673-7.

111.Tasanarong A, Vohakiat A, Hutayanon P, and Piyayotai D. New strategy of α- and γ-tocopherol to prevent contrast-induced acute kidney injury in chronic kidney disease patients undergoing elective coronary procedures. Nephrol Dial Transplant. (2013) 28: 337-44.

112.Toso A, Maioli M, Leoncini M, Gallopin M, Tedeschi D, Micheletti C, et al. Usefulness of atorvastatin (80 mg) in prevention of contrast-induced nephropathy in patients with chronic renal disease. Am J Cardiol. (2010) 105: 288-92.

113.Uyarel H, Çam N, Ergelen M, Akkaya E, Ayhan E, Işık T, et al. Contrast-induced nephropathy in patients undergoing primary angioplasty for acute myocardial infarction: incidence, a simple risk score, and prognosis. (2009).

114.Valappil SP, Kunjukrishnapillai S, Iype M, Koshy AG, Viswanathan S, Gupta PN, et al. Predictors of contrast induced nephropathy and the applicability of the Mehran risk score in high risk patients undergoing coronary angioplasty-A study from a tertiary care center in South India. Indian Heart J. (2018) 70: 399-404.

115.Valappil SP, Kunjukrishnapillai S, Viswanathan S, Koshy AG, Gupta PN, Velayudhan RV, et al. Remote ischemic preconditioning for prevention of contrast induced nephropathy-Insights from an Indian study. Indian Heart J. (2018) 70: 857-63.

116.Vasheghani-Farahani A, Sadigh G, Kassaian SE, Khatami SM, Fotouhi A, Razavi SA, et al. Sodium bicarbonate plus isotonic saline versus saline for prevention of contrast-induced nephropathy in patients undergoing coronary angiography: a randomized controlled trial. Am J Kidney Dis. (2009) 54: 610-8.

117.Webb JG, Pate GE, Humphries KH, Buller CE, Shalansky S, Al Shamari A, et al. A randomized controlled trial of intravenous N-acetylcysteine for the prevention of contrast-induced nephropathy after cardiac catheterization: lack of effect. Am Heart J. (2004) 148: 422-9.

118.Weisbord SD, Gallagher M, Jneid H, Garcia S, Cass A, Thwin SS, et al. Outcomes after Angiography with Sodium Bicarbonate and Acetylcysteine. N Engl J Med. (2018) 378: 603-14.

119.Weisbord SD, Hartwig KC, Sonel AF, Fine MJ, and Palevsky P. The incidence of clinically significant contrast-induced nephropathy following non-emergent coronary angiography. Catheter Cardiovasc Interv. (2008) 71: 879-85.

120.Xu ZR, Chen J, Liu YH, Liu Y, and Tan N. The predictive value of the renal resistive index for contrast-induced nephropathy in patients with acute coronary syndrome. BMC Cardiovasc Disord. (2019) 19: 36.

121.Yang SC, Fu NK, Zhang J, Liang M, Cong HL, Lin WH, et al. Preventive Effects of Alprostadil Against Contrast-Induced Nephropathy Inpatients With Renal Insufficiency Undergoing Percutaneous Coronary Intervention. Angiology. (2018) 69: 393-99.

122.Yoon HJ, and Hur SH. Determination of safe contrast media dosage to estimated glomerular filtration rate ratios to avoid contrast-induced nephropathy after elective percutaneous coronary intervention. Korean Circ J. (2011) 41: 265-71.

123.Zhou F, Song W, Wang Z, Yin L, Yang S, Yang F, et al. Effects of remote ischemic preconditioning on contrast induced nephropathy after percutaneous coronary intervention in patients with acute coronary syndrome. Medicine (Baltimore). (2018) 97: e9579.
